# ROS-responsive hydrogel-delivered miR-665 targets STAT3 to alleviate inflammation and promote hair follicle regeneration in alopecia areata

**DOI:** 10.1186/s12951-026-04214-7

**Published:** 2026-02-21

**Authors:** Wenrong Luo, Wenjing Tantai, Qixiang Gui, Xiaohai Zhu, Zheyuan Hu, Xiang Jie, Zhiwan Liu, Yufei Li, Jiansheng Zheng, Lie Zhu, Minjuan Wu

**Affiliations:** 1 Department of Burns and Plastic Surgery, The Second Affiliated Hospital of Naval Medical University, 450 Fengyang Road, Huangpu District, Shanghai, China; 2 Department of Histology and Embryology, Naval Medical University, 800 Xiangyin Road, Yangpu District, Shanghai, China; 3 Department of Plastic Surgery, Shanghai East Hospital, Tongji University School of Medicine, Shanghai, China; 4 Department of Burns and Plastic Surgery, The 909th Hospital, School of Medicine, Xiamen University, 269 Zhanghua Middle Road, Xiangcheng District, Zhangzhou, China

**Keywords:** Hydrogel, MiR-665, Alopecia areata, STAT3, ROS-responsive delivery

## Abstract

**Background:**

Alopecia areata (AA) is an autoimmune disorder characterized by γ-interferon (IFN-γ)-driven CD8 + T-cell infiltration and overactivation of the JAK-STAT pathway; however, safe and long-acting therapies are lacking. MicroRNA (miRNA)-based interventions hold promise as alternatives, but their clinical translation is hindered by poor stability and the absence of targeted delivery systems.

**Methods:**

We identified miR-665 as a key regulator of STAT3 in embryonic mesenchymal stem cell–derived extracellular vesicles via RNA sequencing and functional screening. An injectable, reactive oxygen species (ROS)-responsive hydrogel (PVA-TSPBA) was developed to enable localized and sustained delivery of miR-665. The physicochemical properties, miRNA release kinetics, and biocompatibility of the hydrogels were systematically characterized. Therapeutic efficacy was evaluated in an imiquimod-induced AA mouse model through macroscopic, histological, and immunohistochemical analyses.

**Results:**

The PVA-TSPBA hydrogel exhibited excellent injectability, ROS-dependent degradation, and sustained release of miR-665. In vitro, miR-665 overexpression counteracted the IFN-γ–induced suppression of proliferation and migration in keratinocytes and dermal papilla cells by inhibiting STAT3 phosphorylation. In vivo, injection of PVA-TSPBA@miR-665 hydrogel resulted in prolonged miRNA retention, and significantly promoted hair regeneration, restored follicular structure, and reduced T-cell infiltration compared with the control groups.

**Conclusions:**

We developed a biocompatible, ROS-responsive hydrogel platform for the local delivery of miR-665, which effectively attenuated inflammatory signaling and stimulated hair follicle regeneration in AA. This study provides a novel miRNA–biomaterial combination strategy that holds promise for targeted, durable, and safe treatment of AA.

**Graphical abstract:**

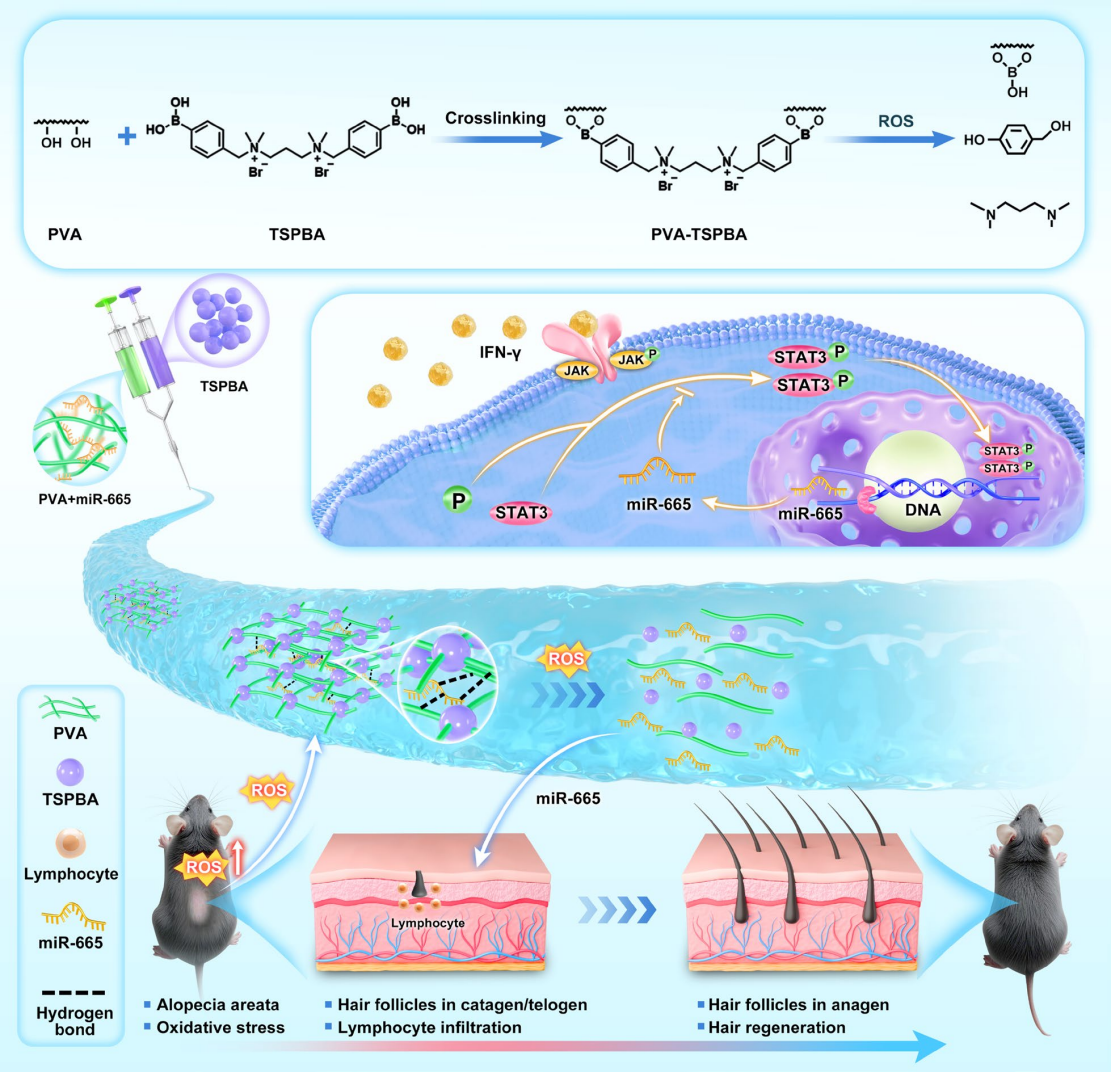

**Supplementary Information:**

The online version contains supplementary material available at 10.1186/s12951-026-04214-7.

## Introduction

Alopecia areata (AA) is primarily driven by CD8 + T cells that secrete γ-interferon (IFN-γ); this cytokine overactivates Janus kinase-signal transducer and activator of transcription (JAK-STAT) signaling, thereby disrupting the immune privilege of hair follicles and triggering follicular dystrophy and hair cycle arrest [1, 2]. Current treatment strategies for AA—including corticosteroids, JAK inhibitors, and monoclonal antibodies—provide only transient relief and suffer from multiple severe limitations, such as systemic immunosuppression, dose-dependent toxicity, and high recurrence rates [3–6]. Long-term administration of JAK inhibitors is associated with increased risks of acne, major adverse cardiovascular events, venous thromboembolism, malignancy, serious infections, and all-cause mortality [4, 5], whereas intralesional steroid injections induce localized skin atrophy, injection-site pain, and hypopigmentation [3]. These limitations underscore the need for targeted therapies that restore local immune homeostasis without compromising systemic balance.

Notably, microRNAs (miRNAs) are a class of small non-coding RNA molecules (19–23 nucleotides in length) that participate in the posttranscriptional regulation of gene expression [7]. The dysregulation of miRNAs in autoimmune diseases has been well established, and miRNAs have been validated as diagnostic biomarkers, prognostic tools, and novel therapeutic targets [8–11]. These findings provide a theoretical basis for miRNA-based interventions in AA. Specifically, we identified miR-665 in extracellular vesicles (EVs) derived from human embryonic mesenchymal stem cells (EMSC-EVs), which can promote hair regeneration in AA by targeting STAT3. Additionally, miR-665 has been confirmed to target STAT3 in multiple tumor types, where it modulates tumor cell proliferation and migration [12, 13]. Conversely, despite their clinical translation potential, miRNA-based therapies are hindered by inherent challenges, including rapid enzymatic degradation in vivo, off-target effects, and transient bioavailability [7, 14]. Traditional delivery systems such as liposomes and viral vectors are further limited by poor targeting, inadequate stability, and immunogenicity [15, 16]. Therefore, there is a pressing need for vector systems capable of sustaining miRNA activity and enabling localized, on-demand release [17].

The AA microenvironment is characterized by elevated reactive oxygen species (ROS) levels, which create an exploitable niche for stimuli-responsive drug delivery [18]. Notably, an injectable hydrogel (PVA-TSPBA) was engineered by crosslinking polyvinyl alcohol (PVA) with N^1^, N^3^-bis(4-boronobenzyl)-N^1^, N^1^, N^3^, N^3^-tetramethylpropane-1,3-diaminium bromide (TSPBA), with miR-665 encapsulated in its matrix. Upon exposure to ROS, the PVA-TSPBA hydrogel undergoes borate bond cleavage, which releases miR-665 [19, 20]. This design confers dual advantages: [1] the porous architecture protects miRNAs from nuclease degradation while mimicking the three-dimensional (3D) network structure of the extracellular matrix [21, 22]; [2] the ROS-responsive release of miR-665 is triggered on demand within AA lesions. By synergizing the immunomodulatory properties of miRNAs with the delivery capabilities of smart biomaterials, this study establishes a precision medicine framework for AA. The experimental design is illustrated in (Graphical abstract).

## Materials and methods

### PVA-TSPBA hydrogel

#### PVA-TSPBA hydrogel synthesis

PVA (molecular weight 30,000–70,000 Da) was purchased from Beyotime (China), and TSPBA was obtained from Weihua Bio (China). PVA-TSPBA hydrogels were prepared by dissolving PVA in deionized water at 60 °C to form a 6% (weight/weight, w/w) solution, while TSPBA was dissolved in deionized water to prepare 2–5% (w/w) solutions. Equal volumes of PVA and TSPBA solutions were mixed rapidly at room temperature; instantaneous gelation occurred upon mixing, without the need for additional stirring or standing [19]. For the miR-665-loaded hydrogels (PVA-TSPBA@miR-665), a recombinant miR-665 mimic was first added to a 6% (w/w) PVA solution under aseptic conditions. The mixture was gently vortexed (1000 rpm) for 1 min to achieve homogeneous dispersion of miR-665, with mild shear stress to preserve the structural integrity of the miRNA. This miR-665-PVA mixture was then immediately mixed with the TSPBA solution at a 1:1 volume ratio; gelation occurred instantaneously upon mixing, without requiring further stirring or incubation. MiR-665 and miR-665^Cy3^ mimics were obtained from GeneAdv Co., Ltd. (China).

#### Injectable properties

A 2-ml disposable medical syringe was used to inject the hydrogel into phosphate-buffered saline (PBS), and then the hydrogel was removed from PBS and left on filter paper for observation.

#### Hydrogel characterization

For microstructure analysis, the PVA-TSPBA hydrogel was first subjected to freeze-drying via a vacuum freeze dryer (FD-1-50Plus, Boyikang, China) for 48 h to preserve its three-dimensional network structure. The lyophilized sample was sputter-coated with gold (thickness ~10 nm) and observed under a scanning electron microscope (SEM) (Sigma 300, ZEISS, Germany) at an accelerating voltage of 5 kV. ImageJ software was used to quantify the pore size distribution, with at least 50 randomly selected pores measured per sample to calculate the average diameter and distribution range.

Fourier transform infrared spectroscopy (FTIR) was performed using a Nicolet iS 10 spectrometer (Thermo Fisher Scientific, USA) to analyze chemical interactions. The freeze-dried hydrogel was ground into a fine powder and mixed with KBr (1:100, w/w) to form pellets. Spectra were collected in the range of 4000–400 cm⁻¹ with a resolution of 4 cm⁻¹ and 32 scans.

Rheological properties were evaluated via a HAAKE MARS60 rotational rheometer (Thermo Scientific, USA) equipped with a 20 mm parallel plate geometry (gap size = 1 mm) at room temperature (25 °C). Amplitude sweep tests were conducted over a strain range of 0.01–100% at a fixed frequency of 1 Hz. Frequency sweep tests were performed at a fixed strain of 1% over a frequency range of 0.1–100 Hz to assess the dynamic mechanical behavior, with the storage modulus (G’) and loss modulus (G’’) recorded to characterize the elastic and viscous properties, respectively.

To observe cell growth within the hydrogel, Human immortalized keratinocytes (HaCaT cells) were seeded into the PVA-TSPBA hydrogel at a density of 5 × 10⁴ cells/mL and cultured for 10 h. The cell-hydrogel constructs were fixed with 2.5% glutaraldehyde (Sigma-Aldrich, USA) for 2 h at 4 °C, followed by gradient dehydration with ethanol (30%, 50%, 70%, 90%, 100%) and critical point drying. The samples were then observed under a biological cryo-SEM (FEI Quanta 450, Thermo Fisher Scientific, USA) to visualize the cell morphology, adhesion, and distribution within the hydrogel network.

#### Loading efficiency (LE) of the hydrogel

The LE of miR-665 in the PVA-TSPBA hydrogel was determined as follows: (1) Hydrogel preparation: Preparation of PVA-TSPBA@miR-665 hydrogels was performed as previously described. (2) Recovery of unloaded miR-665: Each PVA-TSPBA@miR-665 hydrogel was incubated in 1 mL PBS at 37 °C for 2 h to allow unbound miR-665 to diffuse into the supernatant. The supernatant was collected by centrifugation at 3000 g for 5 min to remove residual hydrogel debris. (3) Quantification of unloaded miR-665: The concentration of miR-665 in the supernatant was measured via an ultramicro spectrophotometer (Hengmei, China) at an absorbance wavelength of 260 nm, with background correction at 280 nm to eliminate interference from nonnucleic acid components. A standard curve was generated using miR-665 mimics of known concentrations to ensure accurate quantification. (4) Calculation of loading efficiency:

LE was calculated via the following formula:

$$\:LE\left(\mathrm{\%}\right)=\left(\frac{Initial\:amount\:of\:miR-665\:added-Amount\:of\:unloaded\:miR-665\:in\:supernatant}{Initial\:amount\:of\:miR-665\:added}\right)\times\:100$$

#### Hydrogel curves

For degradation curve analysis, lyophilized PVA-TSPBA hydrogels were weighed to record the initial dry weight (W₀), and then immersed in 5 mL PBS or H₂O₂ solutions (0, 1, 10, 100 µM; 0 µM = PBS control) with daily solution refreshment; at predetermined time points (0, 2, 4, 6, 8, 10, 12, and 14 days), the hydrogels were retrieved, blotted, lyophilized, and reweighed (Wₜ), and the degradation ratio was calculated as [(W₀ - Wₜ)/W₀] × 100. For swelling curve analysis, lyophilized hydrogels were weighed (W₀), immersed in 5 mL PBS at 37 °C, retrieved at specified time points (0, 2, 4, 6, 8, 10, 12, 15, 30, 40, and 60 min), blotted to record the wet weight (Wₛ), and the swelling ratio was calculated as [(Wₛ - W₀)/W₀] × 100. For drug release kinetics, a standard curve was generated by diluting bovine serum albumin (BSA, Beyotime, China) to 0, 10, 20, 40, 60, 80, or 100 µg/mL, mixing 200 µL each with 1 mL of Coomassie Brilliant Blue G-250 (Beyotime, China) for 5 min in the dark, measuring the absorbance at 595 nm via a microplate reader (BioTek, USA), and deriving the regression equation; PVA-TSPBA@BSA hydrogels were prepared by adding 0.1 mL of 1500 µg/mL BSA solution to 1 mL 6% w/w PVA, vortexing (1000 rpm, 1 min), mixing with 4% w/w TSPBA at 1:1 v/v, and then immersing in 10 mL H₂O₂ solution (0, 1, 10, 100 µM) at 37 °C; at each time point (0, 2, 4, 6, 8, 10, 12, and 14 days), 0.1 mL supernatant was collected (with 0.1 mL fresh solution replenished), and reacted with Coomassie reagent, the absorbance was measured, the BSA concentration was calculated via a standard curve, and the cumulative release percentage was determined as [(Total released BSA at time t)/Initial encapsulated BSA] × 100.

Given that miRNAs exhibit short-term activity retention in H₂O₂ solution, BSA was used as a model protein to characterize the ROS-responsive and time-dependent release behavior of the hydrogel, which serves as a surrogate for predicting miR-665 release trends.

### Cells, EVs and RNA

#### EVs derived from human umbilical cord mesenchymal stem cell (UMSC-EVs) and EMSC-EVs were isolated

Human umbilical cord mesenchymal stem cells (UMSCs) and embryonic mesenchymal stem cells (EMSCs) were obtained from the Department of Histology and Embryology, Naval Medical University. EMSCs/UMSCs were cultured in DMEM/F12 (Thermo Fisher Scientific, USA) + 10% EVs-depleted fetal bovine serum (Gibco, USA). EVs in the conditioned medium were isolated by differential centrifugation at 300 g for 10 min, 15,000 g for 35 min, and 100,000 g for 75 min.

#### Nanoparticle tracking analysis (NTA) of EVs

The size distribution and particle concentration of isolated EVs were quantified by NTA using a NanoSight NS300 system (Malvern Instruments Ltd., UK). Data acquisition and analysis were performed using NTA software (version 3.4 Build 3.4.4, Malvern Instruments Ltd.) with the Finite Track Length Adjustment algorithm and a detection threshold of 5. Prior to measurement, exosome samples were filtered through 0.22 μm membranes and serially diluted 10–100 fold in PBS to achieve an optimal particle density. The final measured parameters were: particle concentration = 9.49 × 10⁹ ± 5.60 × 10⁸ particles/mL; 23.0 ± 1.0 particles/frame; 27.4 ± 1.1 centres/frame.

#### Transmission electron microscopy (TEM) of EVs

A 30 µL aliquot of EV-suspension was applied onto carbon-coated copper grids placed on Parafilm, and incubated for 2–5 min to allow adsorption. Excess sample solution was gently blotted from the grid edge using a piece of filter paper, followed by air-drying on filter paper for approximately 10 min. Once the support film was sufficiently dried, a drop of uranyl acetate solution was added for negative staining for 90 s. The excess staining solution was then removed by blotting, and the grids were mounted on filter paper and air-dried for 3 h prior to TEM observation.

#### RNA sequencing

EVs RNA was extracted via TRIzol LS (Invitrogen, USA). Libraries were prepared with the NEBNext Small RNA Library Prep Kit (Invitrogen, USA) and sequenced on an Illumina NovaSeq 6000 (2 × 150 bp). Differential miRNA expression was analyzed using DESeq2 with thresholds of |log2 fold change (FC)| > 1 and false discovery rate < 0.05.

#### Lentiviral transfection

HaCaT cells were provided by the Department of Histology and Embryology and Research Center of Developmental Biology, Naval Medical University, and maintained in Dulbecco’s modified eagle medium supplemented with 10% fetal bovine serum and 1% penicillin-streptomycin at 37 °C in a humidified atmosphere containing 5% CO₂. Human dermal papilla cells (DPCs) were obtained from the Meisen CTCC Cell Bank (China) and cultured in DPCs-specific medium (Meisen CTCC) under the same environmental conditions.

Lentiviral vectors encoding miR-665 overexpression (OE), knockdown (KD), or negative control (NC) sequences were constructed by GeneAdv Co., Ltd. (China), with each vector coexpressing a green fluorescent protein (GFP) reporter gene to facilitate transfection tracking. For lentivirus packaging, 293 T cells were seeded in 10 cm dishes at a density of 5 × 10⁶ cells/dish 24 h prior to transfection. The recombinant lentiviral plasmids (10 µg) were cotransfected with packaging plasmids (pMD2.G, 5 µg; psPAX2, 7.5 µg) using Lipofectamine 3000 (Invitrogen) according to the manufacturer’s protocol. Culture supernatants containing lentiviruses were harvested at 48 and 72 h posttransfection, filtered through 0.45 μm cellulose acetate membranes, and concentrated via ultracentrifugation (25,000 g for 2 h at 4 °C). Viral titers were determined via a GFP-based limiting dilution assay, with titers consistently ranging from 1 × 10⁸ to 5 × 10⁸ transducing units (TU)/mL.

For the transfection of HaCaT cells and DPCs, the cells were seeded in 6-well plates at 3 × 10⁵ cells/well and allowed to adhere overnight. Lentiviruses were added at a multiplicity of infection of 10 for HaCaT cells and 15 for DPCs, with 8 µg/mL polybrene (Sigma-Aldrich, Ger) included to increase transduction efficiency. The culture medium was replaced with fresh complete medium 12 h postinfection. Transfection efficiency was evaluated via fluorescence microscopy (Olympus IX73, Jap) at 48–72 h post-transfection, with GFP-positive cells counted in five random fields per well to calculate the percentage of transfected cells (typically > 80% for both cell types).

In the rescue experiment, plasmids encoding STAT3 overexpression were constructed by GeneAdv Co., Ltd. (China) and transfected into the corresponding miR-665 OE HaCaT cells and miR-665 OE DPCs using Lipofectamine^™^ 3000 (Thermo Fisher Scientific, USA) following the manufacturer’s recommended protocol. These cells were designated as miR-665 OE + STAT3 OE.

### AA model

#### In vitro AA model

Recombinant human IFN-γ (16.88 kDa) was purchased from Novoprotein (China). IFN-γ was dissolved in PBS and then added to complete medium. HaCaT cells and DPCs were cultured with the above media.

#### In Vitro hair follicle culture

Healthy hair follicles were obtained from adult male volunteers with intact scalp health who attended the Department of Burns and Plastic Surgery, The Second Affiliated Hospital of Naval Medical University; all volunteers provided written informed consent, in compliance with institutional ethical guidelines.

All reagents used in this process were purchased from Beyotime (China), unless otherwise specified. For culture medium preparation: Recombinant human insulin was dissolved in 1% dilute acetic acid to a 10 mg/mL stock solution, and hydrocortisone was dissolved in 1% absolute ethanol to a 10 mg/mL stock solution (subsequently diluted in William’s E medium with L-glutamine (Procell, China) to a 10 µg/mL working solution). The complete hair follicle medium was formulated as follows: 45 mL William’s E medium, 50 µL 10 mg/mL recombinant human insulin, 50 µL 10 µg/mL hydrocortisone, 5 mL serum, and 500 µL 1% penicillin-streptomycin solution. For inflammation-induced culture, IFN-γ was added to the complete medium to a final concentration of 100 ng/mL; miRNAs used in each treatment group were chemically synthesized by GeneAdv Co., Ltd.

Under sterile conditions, hair follicles were isolated using sterile forceps and scissors, their hair shafts trimmed to a uniform length, and rinsed repeatedly with PBS containing 1% penicillin-streptomycin. Processed follicles were placed into a 24-well plate, which was inverted and incubated in a cell culture incubator (37 °C, 5% CO₂) for 2 h. After pre-incubation, 0.5 mL of the complete medium was added to each well, and the plate was returned to the incubator for continued culture. Follicle morphology was observed using an upright microscope (Leica, Germany) during the culture period.

#### AA mouse model

5% imiquimod (IMQ) cream was procured from Youshili (China). Male C3H/HeJ mice (8 weeks old, ~ 34 g) were obtained from the Animal Laboratory Center of Naval Medical University and subjected to topical application of IMQ cream on the dorsal neck region. Each application consisted of 50 mg of IMQ cream, which was administered over a circular area with a diameter of approximately 2 cm, twice daily for a duration of 6 weeks to induce AA. After successful AA model induction, skin tissues from the AA lesions were harvested, and hematoxylin and eosin (H&E) staining was performed to assess pathological features of AA, including lymphocyte infiltration, hair follicle dystrophy, and epidermal hyperplasia.

### Animal experiments

#### EVs in vivo administration and efficacy evaluation in AA mice

AA mice were randomly assigned to two groups (*n* = 6 per group): EMSC-EVs group and UMSC-EVs group. For in vivo tracking, EMSC-EVs were labeled with PKH67 fluorescent dye (Beyotime, China) as follows: 100 µg of EVs were resuspended in 1 mL of Diluent C, mixed with 4 µL of 2 × 10⁻⁶ M PKH67 dye, and incubated at room temperature for 5 min. The reaction was quenched by adding an equal volume of EV-depleted fetal bovine serum, and unbound dye was removed via ultracentrifugation at 100,000 g at 4 °C for 75 min; the labeled EMSC-EVs were finally resuspended in PBS. Mice were anesthetized via intraperitoneal injection of chloral hydrate (Beyotime, China), followed by intradermal/subcutaneous injection of 100 µL PBS containing 50 µg EVs into each mouse in the EMSC-EVs and UMSC-EVs groups using a 30G needle at multiple injection sites. Administration was repeated every 7 days after the initial injection. Photographs of alopecia lesions were captured every 6 days to monitor hair regrowth; on day 15, hair density and hair shaft thickness were quantified using a hair lens (Zhoupu, China).

At 24 h after the first injection of PKH67-labeled (Beyotime, China) EMSC-EVs, 3 mice per group were euthanized, and frozen sections of skin from the injection site were prepared. After nuclear labeling with 4’,6-diamidino-2-phenylindole (Beyotime, China), EMSC-EVs localization in hair follicles was observed under a confocal microscope.

#### Screening of candidate miRNAs in AA mice

Candidate miRNAs (including miR-665, miR-487a-3p, miR-539-3p, miR-2355-3p, miR-1185-1-3p, miR-4791, and miR-1304-3p) for in vivo screening were chemically synthesized by GeneAdv Co., Ltd. (China), with their nucleotide sequences provided in Table 1. Prior to miRNA administration, AA mice were anesthetized via intraperitoneal injection of chloral hydrate, and each candidate miRNA was formulated as a sterile preparation by dissolving 5 nmol of the miRNA in 100 µL of nuclease-free water, followed by multi-point intradermal/subcutaneous injection into the dorsal neck alopecia lesions. AA mice were randomly divided into seven groups (*n* = 3 mice per group). Injections were repeated every 7 days for a total treatment period of 28 days, and macroscopic hair regrowth was monitored and documented with a digital camera every 6 days.

**Table 1:** MiRNA nucleotide sequences

miRNA	Nucleotide sequence
miR-665	5‘-ACCAGGAGGCUGAGGCCCCU-3’
miR-487a-3p	5‘-AAUCAUACAGGGACAUCCAGUU-3’
miR-539-3p	5‘-AUCAUACAAGGACAAUUUCUUU-3’
miR-2355-3p	5‘-AUUGUCCUUGCUGUUUGGAGAU-3’
miR-1185-1-3p	5‘-AUAUACAGGGGGAGACUCUUAU-3’
miR-4791	5‘-UGGAUAUGAUGACUGAAA-3’
miR-1304-3p	5‘-UCUCACUGUAGCCUCGAACCCC-3’

#### Hydrogel animal experimental procedures

AA mice were anesthetized via intraperitoneal injection of chloral hydrate, followed by intradermal/subcutaneous injections of two formulations: 100 µL of PVA-TSPBA@miR-665^Cy3^ hydrogel, and 100 µL of aqueous solution containing 5 nmol of free miR-665^Cy3^. Imaging was performed at predefined time points using a multifunctional imaging system (Bio-Rad, USA) for documentation.​.

Following the successful induction of AA, the mice were randomly assigned to four experimental groups, each receiving a distinct treatment: [1] PVA-TSPBA@miR-665^OE^ hydrogel; [2] PVA-TSPBA@miR-665^KD^ hydrogel; [3] PVA-TSPBA@miR-665^NC^ hydrogel; and [4] PVA-TSPBA hydrogel alone. Treatments were initiated at 6 weeks post-IMQ induction, which was designated day 0 of the treatment phase. Each lesion was administered an intradermal/subcutaneous injection of 100 µL hydrogel containing 5 nmol of miR-665 via a 30G needle. Injections were delivered in a grid pattern at a density of 5 injection points per cm² to ensure uniform distribution within the intradermal/subcutaneous layer. ​ Based on miR-665 retention data, treatments were repeated every 10 days, resulting in a total of 3 doses administered on days 0, 10 and 20.​ For assessment, standardized photographs were captured every 6 days to document the overall progression. Macroscopic hair regrowth was specifically monitored on days 9, and 18 via a hair lens. Lesion coverage was quantified through ImageJ analysis of the standardized photographs. On days 11 and 24, *n* = 3 mice per group were euthanized, and the skin lesions, spleen, and major organs (heart, liver, lung, and kidney) were harvested. These samples were subjected to histopathological analysis via H&E staining and immunohistochemistry (IHC) analysis of CD3, STAT3, WNT5a, and β-catenin.

### Dual-luciferase reporter assay

HEK293T cells were cotransfected with pmirGLO-STAT3-3’ untranslated region (UTR) (wild-type/mutant. Plasmids were constructed by GeneAdv Co., Ltd. (China)) and miR-665 mimics/inhibitors. Luciferase activity was measured 48 h post-transfection.

### Cellular experiments

#### Cell counting Kit-8 (CCK-8) cell proliferation assay

CCK-8 was purchased from Beyotime, China. HaCaT cells and DPCs transfected with lentivirus were cultured, and the optical density (OD) at 450 nm was measured per the protocol at 0 h, 24 h, and 48 h after the addition of IFN-γ. To evaluate the safety of the PVA-TSPBA hydrogel, HaCaT cells were directly seeded onto the surface of the PVA-TSPBA hydrogel, and the OD at 450 nm was measured at the aforementioned time points.

#### 5-ethynyl-2’-deoxyuridine (EdU) cell proliferation assay

The EdU Kit was purchased from Beyotime, China. HaCaT cells and DPCs transfected with lentivirus were cultured to the logarithmic growth phase. HaCaT cells and DPCs were labeled following the protocol provided with the EdU Kit, then fixed with 4% paraformaldehyde and stained with the click reaction mixture. Finally, cell nuclei were stained with Hoechst 33,342. Fluorescently labeled cells were counted under a fluorescence microscope.

### Molecular experiments

#### Reverse transcription-quantitative polymerase chain reaction (qRT-PCR)

Total RNA was reverse transcribed via the Mipure Cell/Tissue miRNA Kit (Vazyme, China). RNA expression was quantified via ChamQ SYBR qPCR Master Mix (Vazyme, China) on a QuantStudio 5 (Applied Biosystems, USA). The miRNA sequences are listed in Table 1, and the primer sequences are listed in Table 2. miRNAs and primers were purchased from GeneAdv Co., Ltd. (China).

**Table 2:** Primer nucleotide sequences

Primer	Nucleotide sequence
U6 F	5‘-ATACAGAGAAAGTTAGCACGG −3’
U6 R	5‘-GGAATGCTTCAAAGAGTTGTG −3’
miR-665 F	5‘-TCTGCCGGGACATTCAGGAT −3’
miR-665 R	5‘-ACTGGCTCTCCTTGTTCCAAT −3’
β-Actin F	5‘-TCCATCGGAGCCGAAGAAATC −3’
β-Actin R	5‘-GTGTCGGTGGATCAAAGCACA −3’
STAT3 F	5‘-CTGTGTGACACCAACGACCT −3’
STAT3 R	5‘-GGGTTCAGCACCTTCACCAT −3’
U6 RT	5‘-GTCGTATCCAGTGCGTGTCGTGGAGTCGGCAATTGCACTGGATACGAC-3’
miR-665 RT	5‘-GTCGTATCCAGTGCAGGGTCCGAGGTATTCGCACTGGATACGACAGGGGC-3’

#### Western blotting (WB)

Proteins were extracted with RIPA buffer (Beyotime, China) and resolved by 10% SDS-PAGE (Beyotime, China). After transfer to polyvinylidene difluoride membranes, rabbit antibodies against phosphorylated STAT3 (*p*-STAT3) (1∶2000, CST), mouse antibody against stat3 (1∶2000, CST), mouse antibody against β-actin (1∶1000, Beyotime), and mouse antibody against STAT3 (1∶1000, Beyotime) were used. Anti-rabbit antibody (1∶1000, Abmart) and anti-mouse antibody (1∶1000, Abmart) were incubated with the samples, followed by exposure. Membranes were stripped using antibody stripping buffer and re-probed with other primary antibodies until all target proteins were detected. The exposure maps were analyzed by grayscale using ImageJ. The same method was used with a mouse anti-Alix antibody (1∶1000, CST), rabbit anti-CD9 antibody (1∶1000, CST), rabbit anti-CD63 antibody (1∶1000, CST), and mouse anti-TSG101 antibody (1∶1000, CST) for WB identification of EVs.

### IHC and H&E staining

Tissues were immersed in 4% paraformaldehyde for 24 h, embedded in paraffin, and sectioned at 4-µm thickness. For IHC staining: Sections were deparaffinized with xylene and rehydrated through a graded ethanol series. Antigen retrieval was performed by boiling sections in 0.01 M citrate buffer (pH 6.0) for 15 min. Endogenous peroxidase activity was quenched with 3% H₂O₂ for 10 min at room temperature. Nonspecific binding was blocked with 5% normal goat serum in PBS for 30 min. Sections were incubated with primary antibodies overnight at 4 °C, including rabbit anti-CD3 (1:500, CST), rabbit anti-Wnt5a (1:400, CST), mouse anti-β-catenin (1:600, CST), and rabbit anti-STAT3 (1:500, CST), all diluted per the manufacturer’s protocols. Following three washes with PBS, sections were incubated with horseradish peroxidase-conjugated secondary antibodies for 1 h at 37 °C: Anti-rabbit antibody (1:1000, Abmart) for rabbit-derived primary antibodies (CD3, Wnt5a, STAT3) and anti-mouse antibody (1:1000, Abmart) for the mouse-derived anti-β-catenin. Immunoreactivity was visualized using 3,3’-diaminobenzidine substrate, and nuclei were counterstained with hematoxylin. Finally, sections were dehydrated with graded ethanol, cleared with xylene, and mounted with neutral mounting medium.

For H&E staining: Parallel sections were deparaffinized with xylene, rehydrated with ethanol, stained with hematoxylin for nuclei, differentiated with 1% ethanol hydrochloride, stained with eosin for cytoplasm, dehydrated with ethanol, cleared with xylene, and mounted with neutral mounting medium.

### Statistical analysis

The numerical data are presented as the mean ± standard deviations. The statistical analysis involved conducting multiple group comparisons via one-way ANOVA, and performing two group comparisons via Student’s sample t-test with SPSS 26.0 software (IBM, NY, USA). The results were then visualized via GraphPad Prism software (version 9.0, La Jolla, CA). P values < 0.05 were considered statistically significant.

## Results

### Screening of hair regeneration-promoting miRNAs from EMSC-EVs

**Fig. 1:**
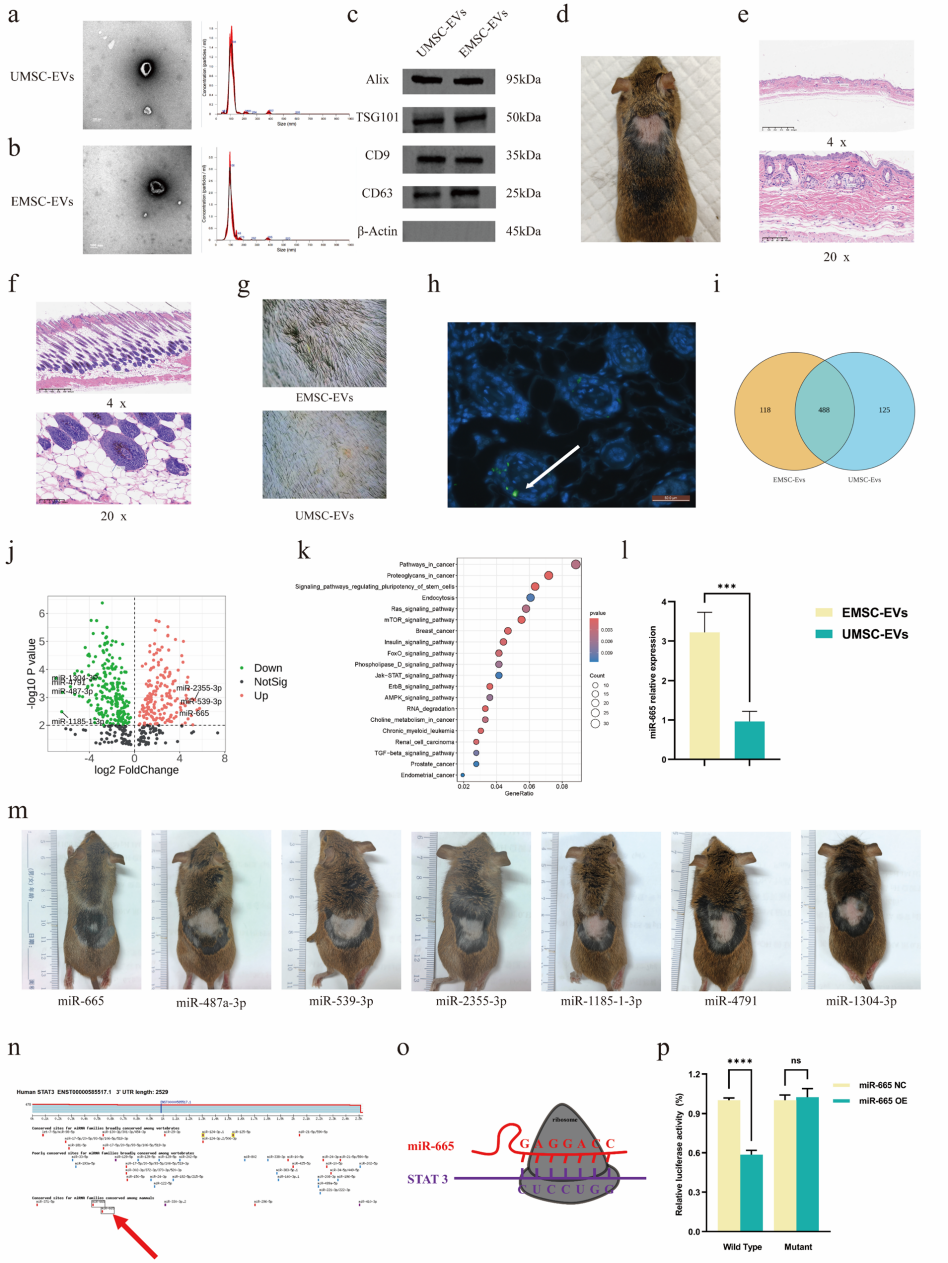
(**a**) Isolation and characterization of UMSC-EVs. (**b**) Isolation and characterization of EMSC-EVs. (**c**) Characterization of UMSC-EVs and EMSC-EVs by WB. (**d**) AA mouse model. (**e**) H&E staining of skin from AA mice. (**f**) H&E staining of skin from healthy mice. (**g**) Hair growth in each group on day 15. (**h**) Tracing results of EMSC-EVs (green fluorescence). (**i**) Venn diagram. (**j**) Volcano plot. **k**. Bubble plot of pathway enrichment analysis. **l**. miR-665 expression in UMSC-EVs and EMSC-EVs. **m**. Preliminary screening of miRNAs. **n**. Target prediction of miR-665 via miRDB database. **o**. Mechanism of miR-665 targeting STAT3 mRNA. **p**. The results of the dual-luciferase reporter assay. (*n* = 3 per group; ns = not significant, ***P < 0.001, ****P < 0.0001)

To identify candidate miRNAs that promote hair follicle regeneration, human EMSC-EVs and human UMSC-EVs were isolated and characterized. TEM showed that both types of EVs exhibited a classic cup-shaped vesicular morphology. NTA indicated that the average size of UMSC-EVs was 100.0 ± 1.7 nm, while that of EMSC-EVs was 104.3 ± 4.4 nm. No significant differences in morphology and size were observed between the two groups of EVs (Fig. 1a, b). WB further demonstrated that both UMSC-EVs and EMSC-EVs expressed the canonical EV markers Alix, TSG101, CD9, and CD63, with no detectable β-actin (Fig. 1c), confirming the purity and suitability of the isolated EVs for subsequent experiments.

**Fig. 2:**
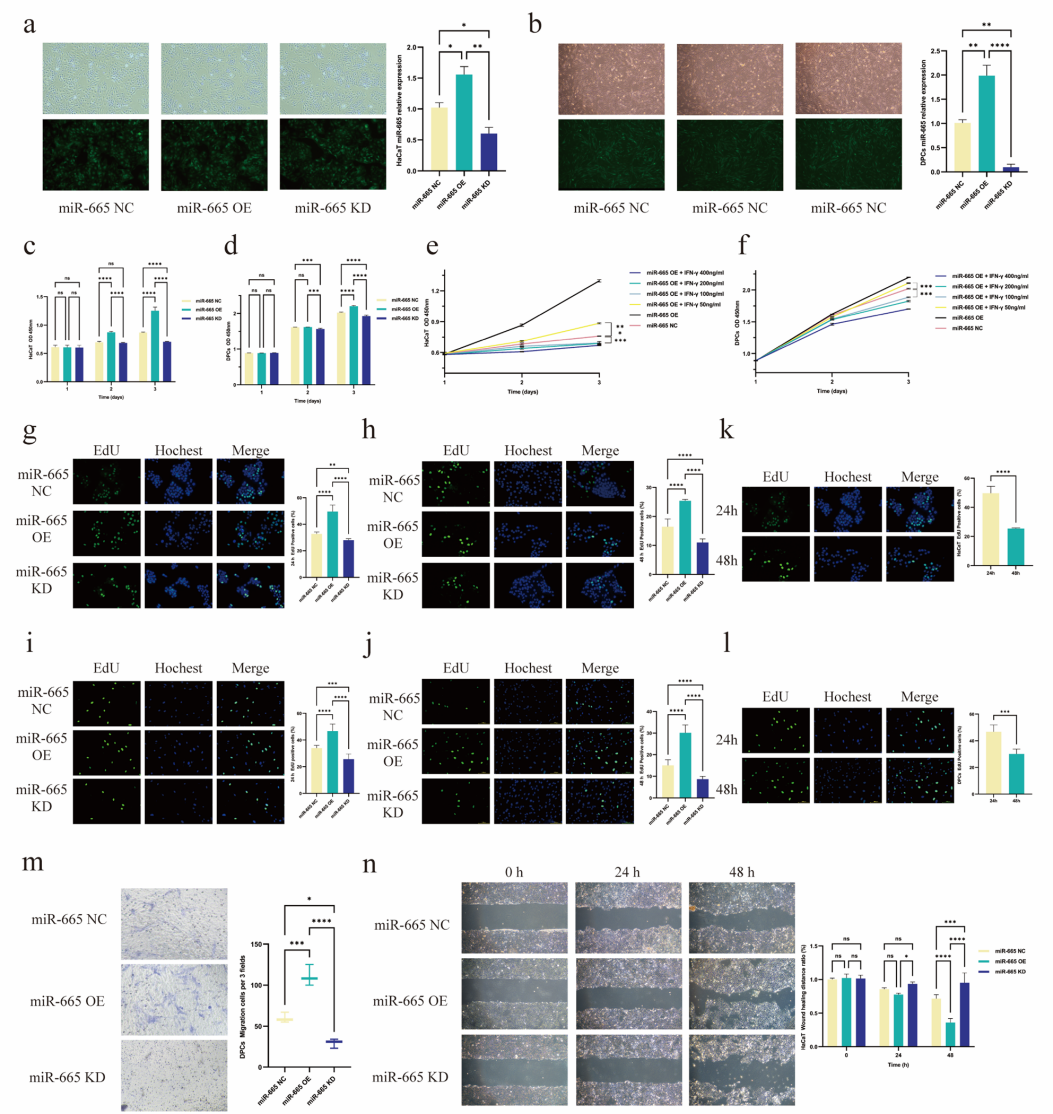
Lentiviral transfection of HaCaT cells (**a**) and DPCs (**b**). Proliferation of HaCaT cells (**c**) and DPCs (**d**) after lentiviral transfection. IFN-γ toxicity assays in HaCaT cells (**e**) and DPCs (**f**). EdU proliferation assays of lentivirus-transfected HaCaT cells stimulated with IFN-γ for 24 h (**g**) and 48 h (**h**). EdU proliferation assays of lentivirus-transfected DPCs stimulated with IFN-γ for 24 h (**i**) and 48 h (**j**). Effect of miR-665 overexpression on the proliferation of IFN-γ-stimulated HaCaT cells (**k**) and DPCs (**l**) at 24 h and 48 h. Migration of lentivirus-transduced DPCs (**m**) and HaCaT cells (**n**) in response to IFN-γ stimulation. (*n* = 3–6 per group; *P < 0.05, **P < 0.01, ***P < 0.001)

An AA mouse model was established by topical application of IMQ to C3H/HeJ mice, as described by Wu et al. (2022) (Fig. 1d) [23]. Compared with healthy skin, H&E staining revealed lymphocyte infiltration, reduced hair follicle density, hair shaft atrophy, epidermal hyperplasia, and disorganized dermal collagen in AA lesions (Fig. 1e, f). These pathological features are consistent with the pathogenesis of human AA, validating the model’s suitability for subsequent investigations [24]. Equal weights of EMSC-EVs and UMSC-EVs were administered via intradermal/subcutaneous injection on days 0 and 7. Hair lens assessment on day 15 demonstrated superior therapeutic efficacy in the EMSC-EVs group, characterized by denser hair and thicker hair shafts than those in the UMSC-EVs group (Fig. 1g). Additionally, EVs tracking experiments showed that PKH67-labeled EMSC-EVs (green fluorescence in Fig. 2h) localized to hair follicles 24 h postinjection, confirming efficient uptake by hair follicles.

RNA sequencing of EMSC-EVs and UMSC-EVs revealed 613 and 606 miRNAs, respectively, with 488 miRNAs coexpressed between the two populations (Fig. 1i). Volcano plot analysis (Fig. 1j) highlighted seven miRNAs with the most significant differential expression, including miR-665, miR-487a-3p, miR-539-3p, miR-2355-3p, miR-1185-1-3p, miR-4791, and miR-1304-3p. Kyoto Encyclopedia of Genes and Genomes pathway enrichment analysis (Fig. 1k) revealed that the JAK-STAT signaling pathway was one of the significantly enriched pathways. Functional screening of these seven miRNAs via intradermal/subcutaneous injection in AA mice (Fig. 1m) demonstrated that miR-665 had the most potent hair follicle regeneration-promoting effect; thus, it was selected as the focus of subsequent experiments. To validate the sequencing data, reverse RT‒qPCR was performed, confirming that miR-665 expression was significantly higher in EMSC-EVs than in UMSC-EVs (Fig. 1l), a result that is consistent with the sequencing data, thereby validating its reliability. miRDB (https://mirdb.org/), a bioinformatics database, predicted that STAT3 is a potential target of miR-665 (Fig. 1n, o). Notably, dual-luciferase reporter assays confirmed that miR-665 directly targets STAT3 (Fig. 1p).

### Establishment and validation of a miR-665 lentiviral transfection model in HaCaT cells and DPCs

To investigate the therapeutic potential of miR-665 in IFN-γ-induced hair follicle dysfunction, systematic in vitro evaluations were performed to assess its effects on HaCaT cells and DPCs. Specifically, plasmids encoding specific miR-665 were first constructed and transfected into 293 T cells; subsequently, lentiviruses harboring the specific miR-665 and GFP genes were harvested, and these specific lentiviruses were then used to transfect HaCaT cells and DPCs. Combined bright-field microscopy, fluorescence imaging, and RT‒qPCR confirmed that the miR-665-specific lentiviruses were successfully transfected into both cell lines, and stable expression was observed (Fig. 2a, b). On the basis of the miR-665 expression levels, these groups were designated the miR-665 NC, miR-665 OE, and miR-665 KD groups. These findings validated the successful establishment of a stable transfection model, which met the criteria for subsequent experiments.

### MiR-665 enhances cellular proliferation and migratory capacity under IFN-γ stress

CCK-8 assays demonstrated that proliferation of HaCaT cells and DPCs was significantly greater in the miR-665 OE group than in the miR-665 NC and KD groups, regardless of exposure to IFN-γ (Fig. 2c, d). To recapitulate the inflammatory microenvironment of AA, an IFN-γ toxicity assay was performed. This assay revealed that when the IFN-γ concentration ranged from 50 to 100 ng/ml, the proliferation rate of cells in the miR-665 OE group was approximately equivalent to that in the miR-665 NC group without IFN-γ stimulation (Fig. 2e, f). To maximize the proliferative disparity between groups, 100 ng/ml was confirmed as the optimal induction concentration for subsequent experiments, which is consistent with findings from previous studies [25, 26].

Furthermore, EdU staining further validated the proliferative advantage of miR-665 overexpression: following exposure of HaCaT cells and DPCs to 100 ng/ml IFN-γ for 24 and 48 h, the rate of DNA synthesis in the miR-665 OE group was significantly greater than that in the miR-665 NC and KD groups (Fig. 2g); notably, the proliferative rates of both cell types at 48 h were significantly lower than those at 24 h (Fig. 2k, l).

Scratch-wound healing assays confirmed that, under IFN-γ stimulation, the migration rate of HaCaT cells in the miR-665 OE group was greater than that in the NC and KD groups (Fig. 2m). Similarly, Transwell assays revealed that the migratory capacity of DPCs in the miR-665 OE group was greater than that in the NC and KD groups (Fig. 2n).

### MiR-665 modulates STAT3 signaling to rescue hair follicle growth in AA Models

**Fig. 3:**
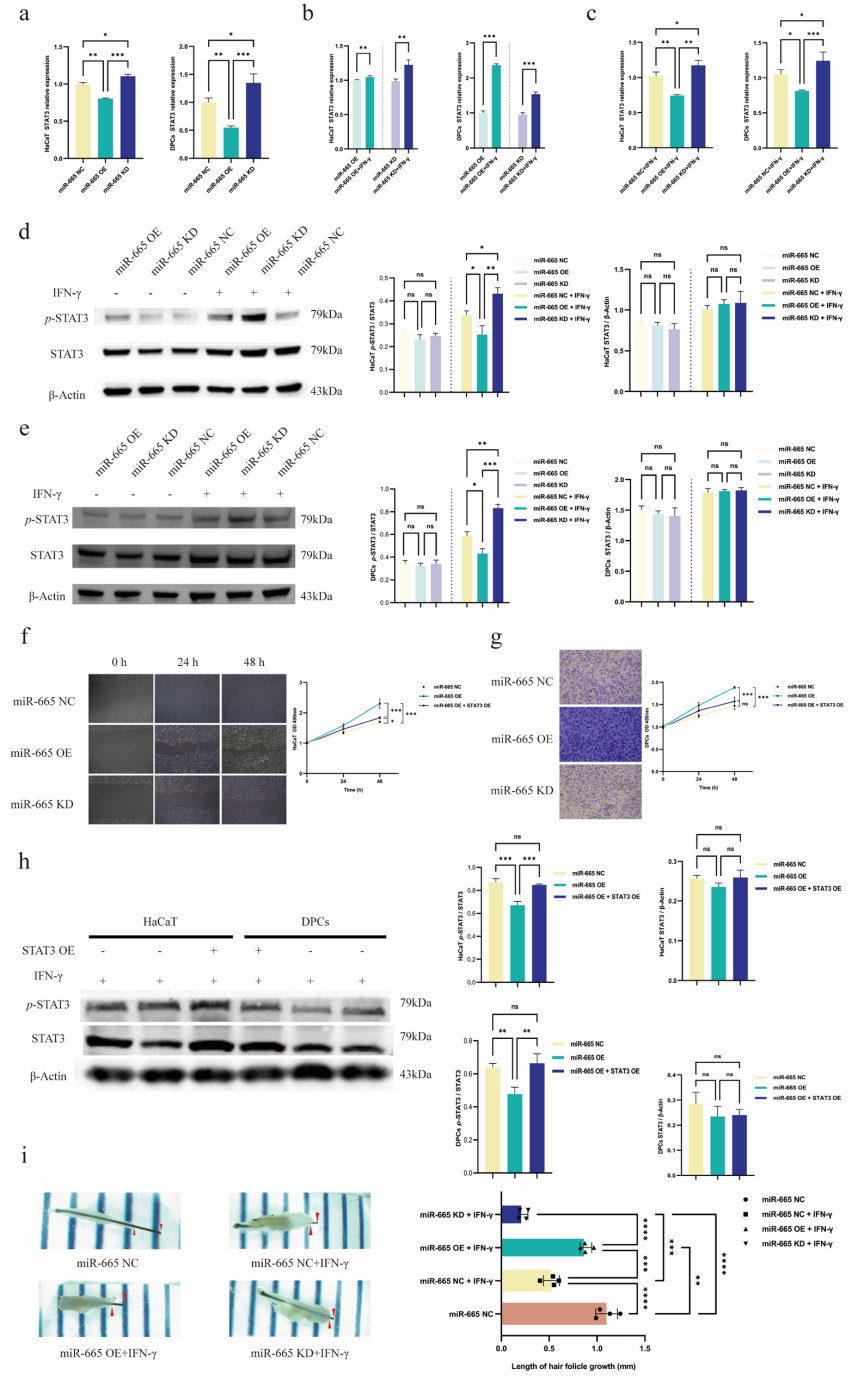
(**a**) STAT3 expression in lentivirus-transfected HaCaT cells and DPCs. (**b**) STAT3 expression in lentivirus-transfected HaCaT cells and DPCs before and after IFN-γ treatment. (**c**) STAT3 expression in IFN-γ-treated HaCaT cells and DPCs. (**d**) WB results showing STAT3 expression in lentivirus-transfected HaCaT cells before and after IFN-γ treatment. (**e**) WB results showing STAT3 expression in lentivirus-transfected DPCs before and after IFN-γ treatment. (**f**) Scratch assay results of HaCaT cells. (**g**) 48 h Transwell assay results of DPCs. (**h**) WB results of rescue experiments in HaCaT cells and DPCs. (**i**) Growth of hair follicles in ex vivo culture on day 5 under different treatment conditions. (*n* = 3–6 per group; *P < 0.05, **P < 0.01, ***P < 0.001)

To elucidate the mechanism by which miR-665 counteracts IFN-γ-induced dysfunction, we examined its effect on STAT3 signaling. RT‒qPCR analysis showed that IFN-γ stimulation increased STAT3 mRNA levels, and miR-665 OE consistently reduced STAT3 mRNA compared to the NC or KD groups, with or without IFN-γ treatment (Fig. 3a). Most notably, Western blot analysis revealed that miR-665 OE significantly suppressed STAT3 phosphorylation (p-STAT3) without altering total STAT3 protein levels (Fig. 3d, e). This indicates that the functional consequence of miR-665 overexpression in this context is the inhibition of STAT3 activation (phosphorylation). The observed dissociation between mRNA reduction and stable total protein may reflect post-transcriptional buffering or protein stability.

To verify that STAT3 is the functional effector mediating miR-665’s effects on hair follicle cells, we performed rescue experiments with three groups: miR-665 NC, miR-665 OE, and miR-665 OE + STAT3 OE. Functional assays showed that miR-665 OE significantly promoted cell migration—with 48 h scratch wound healing rate in HaCaT cells reaching 93.3 ± 3.1%, which was higher than that of the miR-665 OE+STAT3 OE group (57.7 ± 2.5%) and miR-665 NC group (48.7 ± 2.5%, *P* < 0.001; Fig. 3f); and the number of migrated DPCs in Transwell assays (410 ± 36 cells/field in miR-665 OE) was greater than that in the miR-665 OE+STAT3 OE (227 ± 22 cells/field) and miR-665 NC groups (155 ± 19 cells/field, *P* < 0.001; Fig. 3g), while this migratory promotion was partially reversed by STAT3 OE. WB analyses of the rescue groups further corroborated these findings: in both HaCaT cells and DPCs, the *p*-STAT3/STAT3 ratio followed the order of miR-665 NC > miR-665 OE+STAT3 OE > miR-665 OE (*P* < 0.01), with no significant differences in total STAT3/β-actin levels between groups (*P* > 0.05; Fig. 3h).

Consistent with these in vitro findings, ex vivo experiments were performed to validate the effect of miR-665 on intact AA hair follicles. Human-derived hair follicles were exposed to 100 ng/ml IFN-γ and cultured in hair follicle medium supplemented with miR-665 OE, KD, or NC constructs. On day 5, quantitative analysis revealed that the average hair follicle growth length in the miR-665 OE + IFN-γ group (0.885 ± 0.061) was significantly greater than that in both the miR-665 KD + IFN-γ (0.230 ± 0.044) and miR-665 NC + IFN-γ (0.524 ± 0.080) groups. However, follicle growth in the miR-665 OE + IFN-γ group remained significantly lower than that in the IFN-γ-untreated miR-665 NC control group (1.099 ± 0.106) (Fig. 3i). This miR-665-mediated salvage effect on hair follicle growth under IFN-γ stimulation is likely attributable to the enhanced proliferative and migratory capacities observed in the cellular models.

### Design and characterization of the PVA-TSPBA hydrogel

#### Synthesis of the PVA-TSPBA hydrogel

**Fig. 4:**
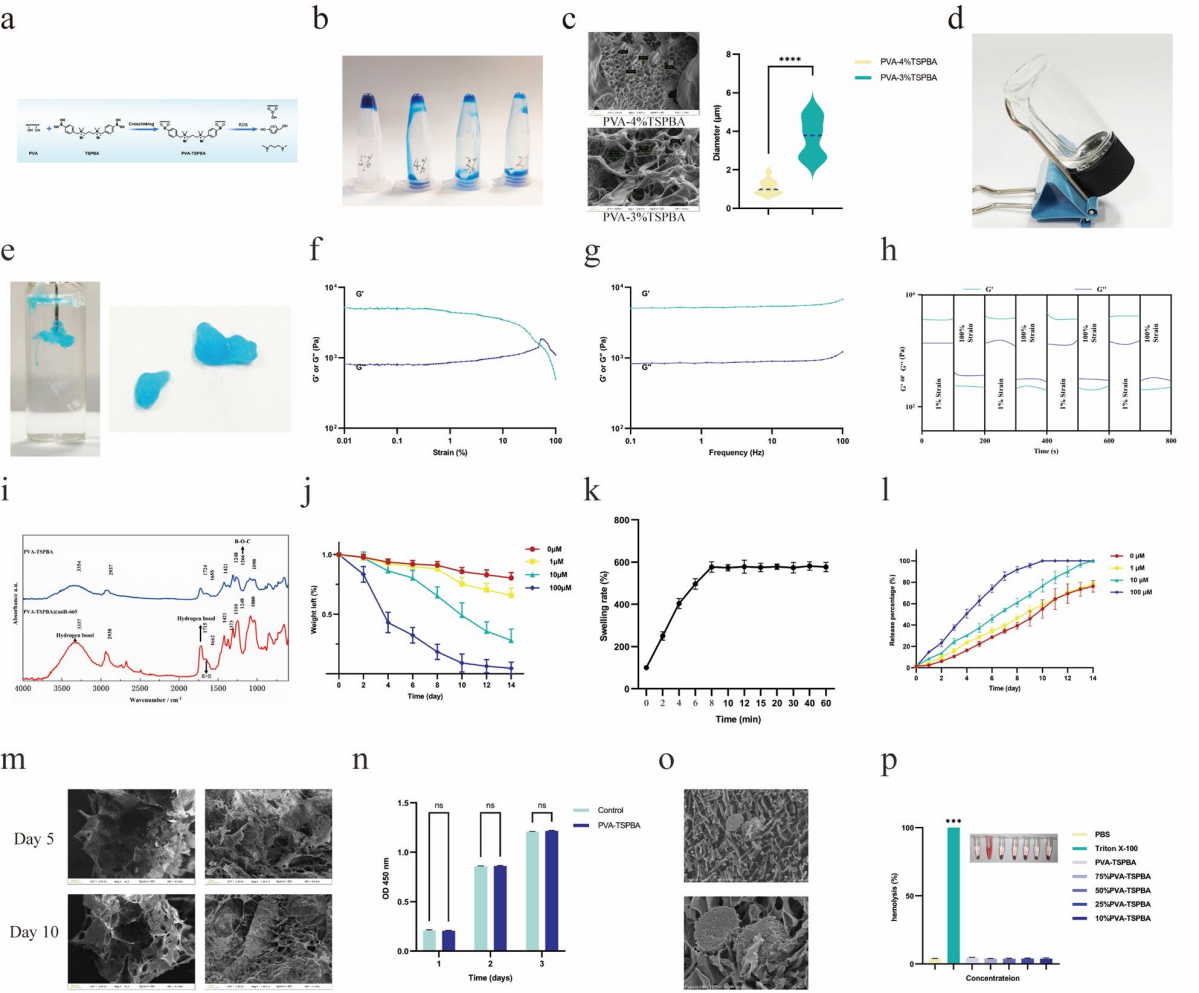
(**a**) Synthesis and degradation of the PVA-TSPBA hydrogel. (**b**) PVA-(2%−5%)TSPBA hydrogel. (**c**) Pore analysis of the PVA-4%TSPBA and PVA-3%TSPBA hydrogels. (**d**) PVA-4%TSPBA hydrogel. (**e**) Injectability and self-healing properties of the PVA-TSPBA hydrogel. (**f**) Strain sweep tests of the PVA-TSPBA hydrogel. (**g**) Frequency sweep tests of the PVA-TSPBA hydrogel. (**h**) Dynamic strain-switching tests of the PVA-4%TSPBA hydrogel. (**i**) The results of FTIR analysis. (**j**) Degradation curves of the PVA-TSPBA hydrogels. **k**. Swelling curves of the PVA-TSPBA hydrogels. **l**. Drug release curves of the PVA-TSPBA hydrogels. **m**. SEM images of PVA-TSPBA hydrogel incubated in PBS on Days 5 and 10. **n**. CCK-8 assay results for the hydrogels. **o**. Cell growth within the hydrogel. **p**. Hemolysis assay results for the hydrogels. (*n* = 10 per group; ns = not significant, ***P < 0.001)

A dynamic network hydrogel was engineered through crosslinking PVA with TSPBA, endowing it with injectability, ROS-responsiveness, and sustained release of miR-665. TSPBA was formulated at concentrations ranging from 2% to 5% (w/w) and individually mixed with a 6% (w/w) PVA solution at a 1:1 volume ratio (Fig. 4a, b). Visual assessment via methylene blue staining demonstrated that the PVA-5%TSPBA hydrogel presented the highest crosslinking density and existed as a solid, whereas the PVA-2%TSPBA hydrogel displayed low crosslinking and remained fully liquid. Combinations of 3% or 4%TSPBA with PVA formed semisolid hydrogels, thereby meeting the fundamental requirements for injectability.

The microstructures of the PVA-4%TSPBA and PVA-3%TSPBA hydrogels were subsequently characterized by SEM. The PVA-4%TSPBA hydrogel exhibited uniform, well-defined pores with a mean diameter of 1.10 ± 0.39 μm (95% confidence interval [CI]: 0.82–1.38 μm), whereas the PVA-3%TSPBA hydrogel displayed irregular pores with a larger average diameter of 3.71 ± 1.09 μm (95% CI: 2.93–4.49 μm) (Fig. 4c). The crosslinking density and porosity are critical determinants of hydrogel degradation kinetics; a higher crosslinking density correlated with a more compact network structure, which typically results in slower degradation [27]. Thus, the PVA-4%TSPBA hydrogel was selected as the vehicle for the delivery of miR-665 for subsequent experiments. This hydrogel is colorless and transparent, exhibiting fluidic solid behavior that maintains structural integrity under resting conditions (Fig. 4d).

#### Injectable capacity and the mechanism of miR-665 loading

The PVA-4%TSPBA hydrogel exhibited excellent injectability, retaining structural integrity and reorganizing into cohesive aggregates upon syringe injection into PBS (Fig. 4e). Strain sweep tests demonstrated that the hydrogel maintained an elastic-dominant state (storage modulus, G’, exceeding the loss modulus, G’’) at strains below ~50%, with G’ intersecting G’’ near this critical strain—indicating resistance to substantial mechanical stress prior to network disruption (Fig. 4f). Notably, frequency sweep tests over 0.1–100 Hz (at 1% strain) revealed that G’ remained significantly greater than G’’ across the entire range, confirming stable mechanical behavior under dynamic physiological frequencies (Fig. 4g). Shear-thinning properties were evident as the viscosity decreased under high shear stress and rapidly recovered at low shear rates. Dynamic strain-switching assays further validated both shear-thinning and self-healing capabilities: under alternating high strain (100%) and low strain (1%), G’ fell below G’’ under high strain but promptly recovered to exceed G’’ upon switching to low strain, demonstrating efficient network reconstitution (Fig. 4h).

FTIR was employed to characterize intermolecular interactions within PVA-TSPBA@miR-665. The characteristic peak at 1266 cm⁻¹ in the PVA-TSPBA hydrogel indicated the formation of borate ester bonds (B-O-C), confirming the dynamic crosslinking between the hydroxyl groups of PVA and borate groups of TSPBA during hydrogel formation. In the PVA-TSPBA@miR-665 hydrogel, a peak shift from 3354 cm⁻¹ (in PVA-TSPBA) to 3337 cm⁻¹ revealed hydrogen bond formation between PVA hydroxyl groups and either the phosphate moieties or nucleobases of miR-665, providing direct evidence for such interactions. Additionally, a peak shift at 1715 cm⁻¹ further supported hydrogen bond formation between the hydrogel matrix and miR-665. Moreover, a peak at 1662 cm⁻¹ implied weak π‒π stacking interactions between the aromatic nucleobases of miR-665 and the bromobenzene rings of TSPBA (Fig. 4i). These interactions collectively enabled stable miR-665 encapsulation without compromising the rheological properties of the hydrogel.

#### Characterization of the swelling, degradation, and drug release behaviors of the PVA-TSPBA hydrogel

To assess the long-term stability and biodegradability of the PVA-TSPBA hydrogel, its degradation profile over a 14-day period was monitored in both PBS and ROS-rich environments (Fig. 4j). The degradation behavior of the hydrogel was both time-dependent and H₂O₂-concentration dependent: specifically, after 14 days, the weight retention rate in the high-ROS environment was approximately 20%, whereas only 10% of the hydrogel had degraded in PBS. To characterize the water absorption capacity of the PVA-TSPBA hydrogel, its swelling kinetics were evaluated. As shown in Fig. 4k, the PVA-TSPBA hydrogel exhibited a rapid initial swelling rate within the first 8 min: the swelling ratio reached approximately 600% at this time point, after which swelling equilibrium was achieved. Additionally, the hydrogel demonstrated a high LE for miR-665, measured at 86.3 ± 4.2%.

Additionally, to investigate the release kinetics from the PVA-TSPBA hydrogel under stimulation with different concentrations of H₂O₂, release profiles were analyzed at H₂O₂ concentrations of 0 µM, 1 µM, 10 µM, and 100 µM (Fig. 4l). The release of miR-665 was both time-dependent and H₂O₂-concentration dependent: by day 10, nearly complete release was observed at the 100 µM H₂O₂ concentration, whereas under PBS, the cumulative amount of released miR-665 was approximately 50% on day 10 and increased to approximately 70% by day 14. Notably, while these release profiles were quantified using BSA as a model protein, the ROS-dependent and time-dependent release trends observed here strongly suggest that miR-665 would exhibit a similar release pattern. This inference is supported by two key observations: [1] Both proteins and miRNAs can bind to PVA via hydrogen bonds, which ensures their stable interactions with the PVA-TSPBA hydrogel matrix [28]; [2] the hydrogel’s degradation (and subsequent cargo release) is driven by ROS-mediated borate bond cleavage—an event that is independent of the encapsulated cargo type. Thus, the BSA release data provide indirect evidence supporting the hypothesis of ROS-responsive, sustained release of miR-665 from the PVA-TSPBA hydrogel. To further confirm the structural alterations associated with degradation in the high-ROS environment, the PVA-TSPBA hydrogel incubated in 10 µM H₂O₂ was subjected to SEM analysis on days 5 and 10. As shown in Fig. 4m, the pore structures within the hydrogel were significantly enlarged, with irregular sizes and a disordered arrangement, accompanied by signs of partial degradation.

#### In vitro biocompatibility and safety evaluation of the PVA-TSPBA hydrogel

**Fig. 5:**
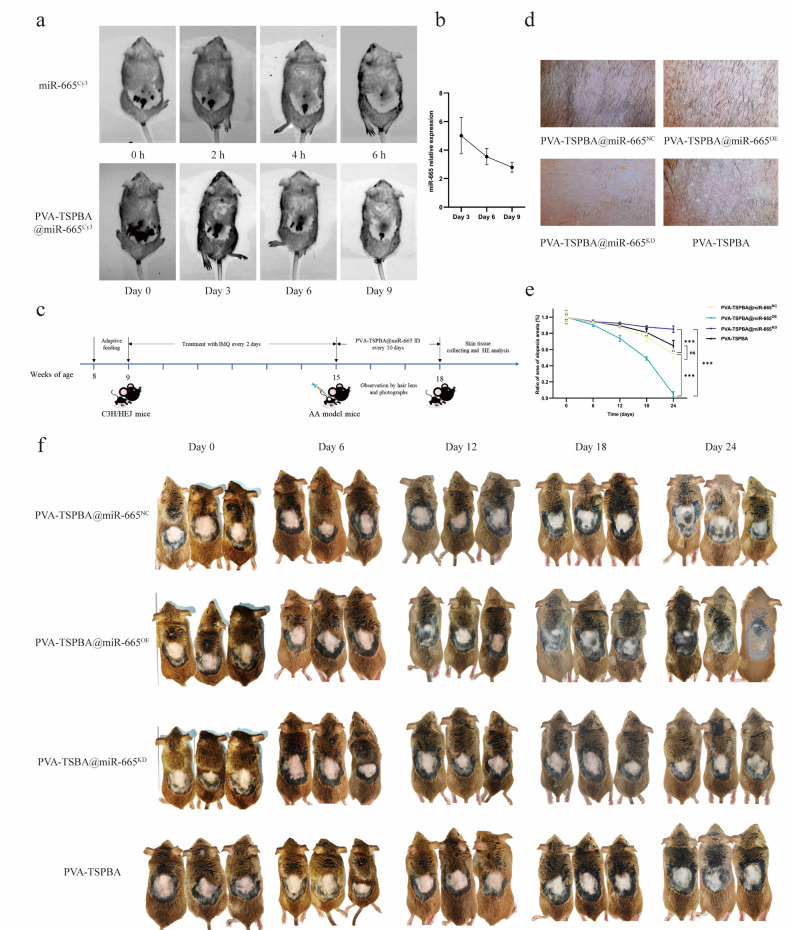
(**a**) In vivo imaging results of the animals. (**b**) miR-665 expression in mouse skin after treatment with the PVA-TSPBA@miR-665 hydrogel. (**c**) Schematic timeline of the therapeutic efficacy experiment. (**d**) Hair growth in each group on day 18. (**e**) Macroscopic hair regrowth in each treatment group over time. (**f**) AA hair regrowth in each treatment group. (*n* = 10 per group; ns = not significant, ***P < 0.001)

Finally, the biocompatibility and safety profiles of the PVA-TSPBA hydrogel were systematically assessed to validate its suitability for biomedical applications. CCK-8 assays were performed to evaluate the effect of the hydrogel on HaCaT cells proliferation, and the results demonstrated that the proliferation rate of HaCaT cells cultured in the presence of the PVA-TSPBA hydrogel was not significantly different from that of the control group (*P* > 0.05), indicating negligible cytotoxicity of the hydrogel (Figs. 4n). Figure 5o illustrates the growth of HaCaT cells within the hydrogel.

To further assess in vitro safety, hemolysis tests were conducted to evaluate potential erythrocyte damage. These tests revealed minimal hemolytic activity: the supernatant remained clear, and the percentage of hemoglobin released was less than 0.5% (Fig. 4p)—a value well within the established safety threshold (< 5%) for biomedical materials. Collectively, these in vitro evaluations confirm that the PVA-TSPBA hydrogel has excellent biocompatibility and favorable safety characteristics, supporting its potential for in vivo applications in hair follicle regeneration.

### Determination of in vivo administration interval based on the miR-665 retention profile

To evaluate the sustained delivery capability and therapeutic potential of the PVA-TSPBA@miR-665 system, miR-665 conjugated with Cy3 (miR-665^Cy3^) was encapsulated into the PVA-TSPBA hydrogel. In vivo imaging provided direct evidence for the sustained delivery capability of the hydrogel. In stark contrast to free miR-665^Cy3^, which was rapidly cleared and became undetectable within 6 h post-injection, the PVA-TSPBA@miR-665^Cy3^ hydrogel maintained a detectable fluorescent signal at the injection site for up to 10 days (Fig. 5a). This > 240-fold extension in local retention relative to the free miRNA control constitutes the primary evidence for ‘sustained delivery’ in this context. To correlate fluorescence signal with biologically available miRNA, RT‒qPCR analyses were performed. The results showed detectable levels of miR-665 in skin tissue at days 3, 6, and 9 post-injection, followed by a gradual decline (Fig. 5b). This kinetic profile is characteristic of hydrogel-based delivery systems: an initial phase of controlled release from the matrix, followed by gradual clearance of the active miRNA, rather than constitutive high-level expression. The 10-day dosing interval was selected based on the conjunction of two criteria: [1] the near-complete loss of fluorescent signal from the hydrogel depot by day 10 (Fig. 5a), indicating depletion of the reservoir; and [2] the RT‒qPCR data showing that functional miR-665 levels remained above a detectable threshold until at least day 9 but were declining, suggesting the need for re-administration to maintain therapeutic activity. The experimental timeline based on this interval is shown in Fig. 5c.

### Therapeutic efficacy of the PVA-TSPBA@miR-665 hydrogel in AA mice

C3H/HeJ mice were topically treated with IMQ to induce AA, followed by intradermal/subcutaneous injection of PVA-TSPBA@miR-665^OE^, PVA-TSPBA@miR-665^KD^, PVA-TSPBA@miR-665^NC^, or the PVA-TSPBA hydrogel alone every 10 days, as outlined in the experimental design (Fig. 5c). On day 18, macroscopic observations revealed that the AA lesioned areas of the mice in the PVA-TSPBA@miR-665^OE^ group presented uniform, high-density hair growth, whereas those in the PVA-TSPBA@miR-665^NC^ group and PVA-TSPBA group presented sparse and irregularly distributed hair; hair was completely absent from the lesioned areas of the PVA-TSPBA@miR-665^KD^ group (Fig. 5d). On day 24, quantitative analysis of lesion coverage demonstrated that the PVA-TSPBA@miR-665^OE^ group achieved 95% lesion coverage (high hair regrowth), while the PVA-TSPBA@miR-665^KD^ group showed minimal recovery (15%). The PVA-TSPBA@miR-665^NC^ group exhibited intermediate partial recovery (45%), and the PVA-TSPBA hydrogel alone group displayed comparable coverage of 36% (Fig. 5e, f).

**Fig. 6:**
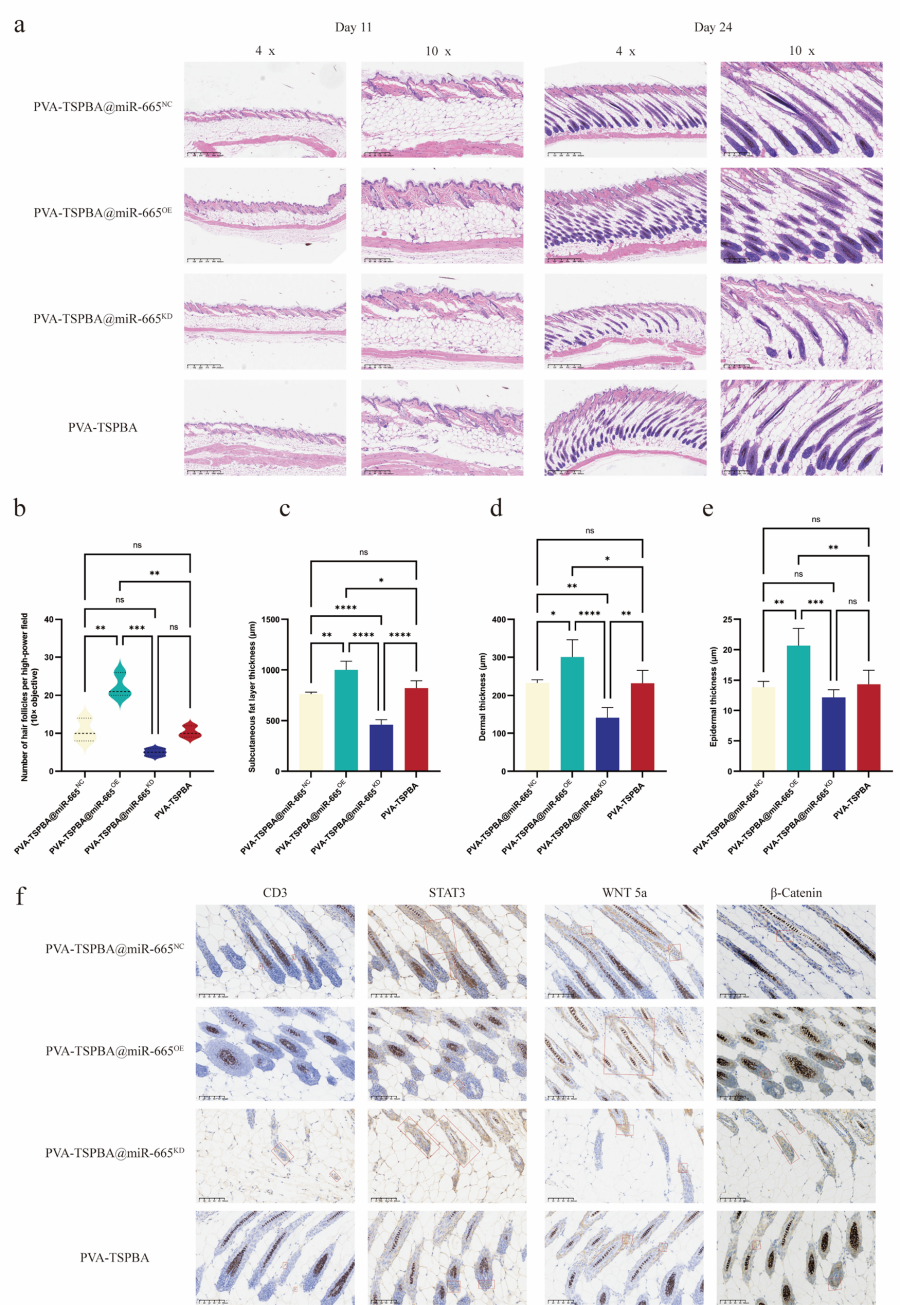
(**a**) H&E staining of skin tissues from animal experiments. (**b**) Quantification of hair follicle numbers in the skin. (**c**) Analysis of subcutaneous fat thickness. (**d**) Analysis of dermal thickness. (**e**) Analysis of epidermal thickness. (**f**) The results of IHC staining of skin tissues from the animal experiments. (*n* = 3 per group; *P < 0.05, **P < 0.01, ***P < 0.001)

H&E staining of skin biopsies collected on days 11 and 24 further corroborated these macroscopic findings (Fig. 6a). On day 11, lymphocyte infiltration in all treated groups (PVA-TSPBA@miR-665^OE^, PVA-TSPBA@miR-665^KD^, PVA-TSPBA@miR-665^NC^, and PVA-TSPBA hydrogel alone) was reduced compared with the AA-associated lymphocyte infiltration pattern observed in Fig. 2e; however, no significant differences in pathological features (including lymphocyte infiltration intensity and skin structural changes) were observed among these four treated groups at this time point.

By day 24, distinct pathological differences emerged across the groups. Skin inflammation in the PVA-TSPBA@miR-665^OE^ group largely subsided, with negligible lymphocyte infiltration detected; hair follicle density notably increased, most follicles were in the anagen phase, and skin layer structures were largely comparable to those of healthy skin (Fig. 6a). In stark contrast, the PVA-TSPBA@miR-665^KD^ group presented persistent focal lymphocyte clusters, low hair follicle density, sebaceous gland atrophy, sweat gland fibrosis, and skin structural abnormalities (epidermal thickening, dermal collagen disruption, and fat layer thinning). Compared with the OE and KD groups, the PVA-TSPBA@miR-665^NC^ and PVA-TSPBA hydrogel alone groups presented comparable intermediate phenotypes, with partial hair follicle recovery, and mild skin structural (Fig. 6b - e). IHC analyses of the four groups further revealed molecular changes (Fig. 6f): CD3 expression (a T lymphocyte marker) around hair follicles was significantly lower in the OE group but than in the KD group. STAT3 expression in hair follicles followed the same trend (markedly decreased in the OE group, with the minimal decreased in the KD group). Additionally, WNT5a and β-catenin—key regulators of hair follicle proliferation—are abundantly expressed in hair follicles and hair shafts, and their expression patterns are consistent with H&E staining-based hair follicle regeneration outcomes.

## Discussion

AA is a common autoimmune alopecia disease, and its pathogenesis involves immune dysregulation and inflammatory damage to hair follicles [1, 2]. Current treatments, including corticosteroids and JAK inhibitors, are limited by their systemic side effects and incomplete efficacy [3–6]. This study developed a novel therapeutic strategy by combining miRNA-based gene regulation with ROS-responsive hydrogels. In this study, miR-665 was identified as a key miRNA that promotes AA hair follicle regeneration in EMSC-EVs, and an injectable PVA-TSPBA hydrogel was developed for continuous delivery of miR-665. In this study, miR-665 was found to alleviate the progression of AA by targeting STAT3 to inhibit the JAK-STAT signaling pathway, while the PVA-TSPBA hydrogel was degraded and locally released miRNA in the ROS environment.

### MiR-665 as a master regulator of hair follicle regeneration

#### EMSC-EVs as a source of therapeutic miRNAs

In the present study, the screening of miR-665 was initiated with a direct comparative assessment of the efficacy of EMSC-EVs and UMSC-EVs in promoting hair regrowth in AA mice. This preliminary comparison revealed that, compared with UMSC-EVs, EMSC-EVs showed superior performance in restoring hair density and accelerating hair follicle regeneration, suggesting that further investigations into the molecular mediators underlying this enhanced activity are needed. Systematic profiling of miRNA cargo within EMSC-EVs was subsequently conducted to identify key regulatory molecules driving their therapeutic effects. Multiple candidate miRNAs isolated from EMSC-EVs were then subjected to intradermal/subcutaneous injection in AA mice for in vivo validation. Through this iterative screening process, miR-665 was ultimately identified as the most potent miRNA in EMSC-EVs, with the strongest capacity to promote hair regrowth in AA mice. These findings—confirming the superiority of EMSC-EVs over UMSC-EVs in AA-associated hair regeneration—align with previous reports documenting the hair follicle regenerative potential of UMSCs, UMSC-EVs, and EMSCs [29–31]. Notably, prior to this study, a direct comparative analysis of the ability of EMSC-EVs and UMSC-EVs to mediate AA-specific hair follicle regeneration was not reported, and the key miRNA (miR-665) responsible for the enhanced efficacy of EMSC-EVs was elucidated—two aspects that collectively represent key innovations of the present work. Prior studies have demonstrated that compared with bone marrow-derived mesenchymal stem cells and adipose-derived mesenchymal stem cells, EMSCs exhibit more potent anti-inflammatory activity. Additionally, these investigations confirmed that both EMSCs and EMSC-EVs exert robust regulatory effects on inflammatory responses [32]. On the basis of these observations, we further hypothesized that EMSC-EVs promote AA-associated hair follicle regeneration through immunomodulation, with potentially greater efficacy than EVs derived from adult stem cells.

RNA sequencing identified that miR-665 was highly expressed in EMSC-EVs, while Kyoto Encyclopedia of Genes and Genomes pathway enrichment analysis further demonstrated significant enrichment of the JAK-STAT signaling pathway. Previous studies have established that the JAK-STAT pathway is a central driver of AA pathogenesis [33], and STAT3 is highly upregulated in AA lesion [34]. These findings suggested a critical mechanistic link between miR-665 and AA pathogenesis. To directly test whether STAT3 is a functional target of miR-665, we performed dual-luciferase reporter assays confirming that STAT3 is a direct target of miR-665; this result is consistent with prior observations in other biological contexts [12, 35]. Importantly, the role of miR-665 in targeting STAT3 within the specific context of hair follicle biology has not been previously documented, representing a potential novel mechanistic insight from the present preclinical study.

Functionally, miR-665 interrupts the IFN-γ-driven inflammatory cascade by targeting STAT3, ultimately alleviating AA pathology. While several JAK inhibitors and cytokine/receptor-targeting monoclonal antibodies have entered clinical practice for AA, there remains an unmet need to develop novel, precisely targeted agents that enhance therapeutic efficacy while minimizing adverse effects [36, 37]. As a naturally occurring miRNA that directly targets STAT3 and modulates JAK–STAT3 signaling, miR-665 represents a promising candidate for precision therapeutics.

#### Rescue of IFN-γ-induced hair follicle dysfunction by miR-665

IFN-γ, a pivotal cytokine in the pathogenesis of AA, drives hair follicle dystrophy and growth cycle arrest via activation of the JAK-STAT pathway, ultimately contributing to AA development [18, 38]. To investigate whether miR-665 could counteract this pathological cascade, in vitro cell models were established. These models revealed that miR-665 OE mitigated the cytotoxic effects of IFN-γ while enhancing the proliferation and migration of HaCaT cells and DPCs—two cell types critical for maintaining hair follicle homeostasis and driving regeneration. Notably, this pro-regenerative role of miR-665 aligns with its reported functions in certain malignancies (e.g., lung cancer) but contrasts with its tumor-suppressive effects in others (e.g., gastric cancer), underscoring the profound context dependency of miRNA biology [39–42].

Our data elucidate that the rescue mechanism centers on the direct targeting and functional modulation of STAT3. The dual-luciferase assay confirmed miR-665 directly binds to the STAT3 3’UTR (Fig. 1p). Functionally, this interaction resulted in a significant inhibition of STAT3 phosphorylation (p-STAT3) upon IFN-γ stimulation, as unequivocally demonstrated by western blot, while total STAT3 protein levels remained comparable across groups (Fig. 3d, e). A concomitant decrease in STAT3 mRNA was observed (Fig. 3a-c). Thus, the primary mechanism supported by our experimental evidence is that miR-665 attenuates the pathogenic JAK-STAT signaling in AA models by suppressing the activation (phosphorylation) of STAT3. The dissociation between reduced STAT3 mRNA and stable total protein is not uncommon in signal transduction, where the phosphorylation state (reflecting activation) can be more readily modulated than the total protein pool, which may exhibit buffering capacity [43, 44].

The therapeutic inhibition of STAT3 in AA underscores its context-dependent role [45]. While constitutive STAT3 activation is a well-established driver of proliferation and migration in diverse cancers, in the autoimmune inflammatory microenvironment of AA, IFN-γ-driven STAT3 hyperactivation functions primarily as a sustaining node in a destructive inflammatory circuit. Therefore, its precise dampening by miR-665 disrupts this core pathogenic pathway, permitting physiological regenerative processes to proceed. Furthermore, literature suggests miR-665 may also influence STAT3 activity through indirect pathways, such as via regulators like NLK or TRIM8 [40, 46]. While these possibilities enrich the mechanistic landscape, our study solidly establishes the functional outcome of reduced STAT3 phosphorylation. Theoretically, the precise modulation of this downstream node contrasts with the broader kinase inhibition profile of JAK inhibitors [49, 50], highlighting a potential avenue for targeted immunomodulation [47, 48]. To further validate the translational relevance, we employed an ex vivo human hair follicle culture system, which better recapitulates the complex tissue microenvironment than monolayer cultures [49]. In this stringent model, miR-665 effectively reversed IFN-γ-induced growth arrest, corroborating its therapeutic potential derived from the cellular mechanism.

In summary, miR-665 counteracts IFN-γ-induced dysfunction by directly targeting STAT3, leading to a significant reduction in its activating phosphorylation. This attenuation of STAT3 signaling underlies the observed rescue of cell proliferation, migration, and ex vivo hair follicle growth, positioning miR-665 as a promising candidate for AA therapy.

### Engineering a smart hydrogel for miRNA delivery

The clinical translation of miRNA therapeutics is hindered by rapid in vivo degradation and the lack of localized, sustained delivery systems [50, 51]. While strategies such as viral vectors, liposomes, and engineered EVs have been developed for miRNA delivery, many of these strategies suffer from poor biocompatibility or unregulated pharmacokinetics [52–55]. Hydrogels offer a promising alternative, as their biomimetic properties and programmable release profiles align with therapeutic needs [56].

Porous hydrogels mimic the native extracellular matrix, encapsulate multiple miRNAs to enable targeted therapy, and possess favorable physicochemical traits (biocompatibility, biodegradability, low toxicity) for loading biological macromolecules (e.g., EVs, proteins, and nucleic acids) [57–60]. Their properties can also be tailored for stimuli responsiveness, expanding the application scope.

#### ROS-responsive degradation and dual-mode controlled release of PVA-TSPBA hydrogel

Chronic inflammation in AA elevates ROS levels, indicating that ROS is a trigger for “smart” hydrogel drug delivery [61, 62]. TSPBA, an ROS-responsive cross-linker, was incorporated into PVA hydrogels: it enhances stability/activity and drives hydrogel degradation via borate ester bond cleavage under high-ROS conditions [63]. When hydrogels were fabricated with varying PVA-TSPBA concentrations, PVA-4%TSPBA was identified as optimal—its high crosslinking density and uniform pores (1.0971 ± 0.3917 μm) ensure miRNA retention, moderate release, and injectable mechanical stability, highlighting its structural-functional relevance in nanoscale design.

The release kinetics of miR-665 from PVA-TSPBA hydrogels are governed by two complementary pathways, as supported by FTIR analysis confirming that miR-665 encapsulated via hydrogen bonding and π–π stacking—interactions that ensure miRNA protection while enabling controlled liberation. Notably, these intermolecular forces contribute to the high loading efficiency of miR-665 within the hydrogel matrix, measured at 86.3 ± 4.2%, ensuring sufficient encapsulation of the therapeutic cargo to sustain biological activity over time. (1) ROS-triggered on-demand release: High H₂O₂ (a surrogate for ROS) accelerates hydrogel disintegration and the burst release of miR-665, which targets inflamed AA lesions. (2) Hydrolysis-mediated sustained release: Release profiles validated this behavior: nearly complete release at 100/10 µM H₂O₂ (day 14) and > 70% release at 1 µM H₂O₂/PBS. This sustained release profile in an oxidative stimulus-free environment (PBS) is an inherent feature of dynamic covalent boronic ester bonds, which undergo gradual hydrolysis in aqueous media. This controlled, slow release is pivotal for maintaining therapeutic drug levels over an extended period, thereby supporting the feasibility of reducing the frequency of drug administration. This integration—high loading efficiency enabled by specific molecular interactions, coupled with dual-mode release kinetics—not only enhances miRNA stability but also supports controlled release kinetics, which may facilitate gradual in vivo miRNA release and contribute to sustained therapeutic effects observed in our AA mouse model [64, 65].

ROS-responsive biomaterials have shown promise in inflammatory disease delivery, and the injectability, shear-thinning, and self-healing properties of PVA-TSPBA hydrogel ensure rapid lesion targeting, stable in vivo retention. Notably, in vivo enzymatic activity and dynamic ROS may alter hydrogel degradation kinetics; real-time in vivo imaging is needed to define optimal dosing intervals. The minimally invasive delivery of the hydrogel improves its clinical applicability, while its modularity enables codelivery of anti-inflammatory agents for synergistic AA therapy. Overall, PVA-TSPBA hydrogel bridges biomaterial engineering and miRNA immunomodulation, addressing key limitations of current AA therapies to offer a precision treatment paradigm.

#### Biocompatibility and safety of the PVA-TSPBA hydrogel

Biocompatibility and safety are paramount for the clinical translation of hydrogel-based therapeutics [66]. The PVA-TSPBA hydrogels exhibited negligible hemolytic activity (< 0.5%) and no cytotoxicity, satisfying the biocompatibility criteria required for drug delivery applications.

FTIR analysis confirmed the formation of hydrogen bonds between miR-665 and the PVA-TSPBA matrix, which contributed to the stable retention of miRNAs within the hydrogel [67]. Compared with covalent conjugation, hydrogen bonding is a weak, reversible interaction—enabling more facile release of miR-665 from the hydrogel matrix [68]. Importantly, such noncovalent interactions do not alter the chemical composition of RNA or compromise its biological activity. Furthermore, compared with liposomes, PVA-TSPBA hydrogels exhibit superior storage stability and reduced immunogenicity relative to viral vectors [15, 16]. Composed of PVA and TSPBA, the hydrogel matrix benefits from the well-characterized safety profile of PVA (a clinically approved synthetic polymer), whereas TSPBA—acting as a chemical cross-linker—generates no toxic byproducts during cross-linking with PVA. In vivo safety was validated through intradermal/subcutaneous injection of PVA-TSPBA@miR-665 into the dorsal skin of mice via a sterile syringe. Throughout the observation period, no cutaneous abnormalities were observed: specifically, no overt erythema, rash, or allergic responses were detected. Moreover, all the mice survived until the experimental endpoint (24 days). These findings support the initial in vivo safety profile of the PVA-TSPBA@miR-665 hydrogels over a 24-day observation period.

### Therapeutic effect of the PVA-TSPBA@miR-665 hydrogel in AA mice

#### Prolonged retention and targeted treatment

The key feature of the PVA-TSPBA hydrogel is its ability to provide sustained delivery, defined here as significantly prolonged local retention and bioavailability compared to the free miRNA. In vivo imaging unequivocally showed that while free miR-665 was cleared within hours, the hydrogel formulation maintained a local depot for up to 10 days (Fig. 5a). Concomitant RT‒qPCR analysis confirmed the presence of functional miR-665 in the tissue over this period, albeit with a declining concentration profile (Fig. 5b). This pattern aligns with the expected pharmacokinetics of a depot system: controlled release from the hydrogel matrix followed by gradual cellular uptake, action, and clearance—not sustained constant expression. The 10-day dosing interval was therefore strategically chosen to replenish the miRNA reservoir as it neared depletion, thereby maintaining therapeutic pressure. This interval reduces treatment frequency compared to modalities requiring daily or more frequent application, potentially enhancing compliance.

On day 18, hair lens evaluation revealed high-density, uniformly distributed hair in the mice treated with the PVA-TSPBA@miR-665^OE^ hydrogel. By day 24, nearly complete hair follicle regeneration (95% coverage) was achieved in the mice that were administered this hydrogel. H&E staining confirmed normalization of lymphocyte infiltration, hair follicle density, epidermal thickness, collagen fiber alignment, and skin layer architecture, indicating a multidimensional therapeutic effect on AA. This magnitude of efficacy is within the range reported for JAK inhibitors and regulatory T-cell expansion in preclinical AA models [69]. Notably, the localized delivery system minimizes systemic exposure [70], which may contribute to improved therapeutic safety—a feature supported by the absence of overt pathological abnormalities in H&E-stained sections of heart, liver, lung, and kidney tissues isolated from mice in the PVA-TSPBA and PVA-TSPBA@miR-665^OE^ hydrogel groups (Additional file 1). No significant differences or abnormalities were identified between the groups via these analyses, thus providing additional evidence supporting the safety profile of the PVA-TSPBA@miR-665 hydrogel.

Further discussion of dosing interval optimization is critical, as in vivo hydrogel degradation kinetics may deviate from in vitro observations. This discrepancy stems from the complexity of the in vivo microenvironment, including tissue-specific enzymes that may accelerate or modulate hydrogel breakdown, dynamic fluctuations in ROS levels—such as ROS bursts during acute inflammatory flares versus gradual reduction during lesion remission—and cell-mediated clearance processes [71]. These factors collectively induce variability in in vivo hydrogel degradation and miR-665 release rates, thus necessitating careful assessment of the risks associated with dosing interval adjustments.

The extension of the dosing interval beyond 10 days poses nonnegligible risks to therapeutic efficacy. In vitro release data (Fig. 4l) demonstrate that miR-665 is fully released by day 10 under exposure to 100 µM H₂O₂—a surrogate for high ROS levels in active AA lesions. If in vivo ROS concentrations are relatively high—such as in severe or acute AA—miR-665 release rates will increase, resulting in premature depletion of the therapeutic agent prior to the next administration. This reactivation reinitiated the IFN-γ-driven inflammatory cascade, reversing the miR-665-mediated inhibition of STAT3 and ultimately impairing therapeutic outcomes. Conversely, shortening the dosing interval also presents challenges. Although in vivo safety data (Additional file 1) confirm the absence of toxicity at the 10-day interval, more frequent injections may increase two key risks: first, local tissue irritation—including mild inflammation at injection sites—which could exacerbate the already disrupted skin microenvironment in AA and potentially compromise hair follicle regeneration; second, the accumulation of hydrogel degradation products, including PVA and TSPBA metabolites. PVA is clinically approved and has a well-documented safety profile. Notably, comprehensive long-term toxicity data are lacking for TSPBA, and these in vivo cumulative effects remain incompletely characterized.

Importantly, previous studies have confirmed that PVA-TSPBA hydrogel degradation is H₂O₂ concentration-dependent. Based on this in vitro observation, we hypothesize that this property confers a “self-regulating” release profile to the system in vivo: miR-665 release would accelerate in inflamed AA lesions with elevated ROS levels to maintain therapeutic concentrations, whereas release would slow in resolving lesions with reduced ROS levels to prevent excessive drug exposure. This hypothesized self-regulatory mechanism reduces the theoretical need for strict dosing interval adjustments, as it mitigates the risk of underdosing in active lesions—where rapid release compensates for accelerated drug consumption—and overdosing in resolving lesions—where slow release prevents unnecessary accumulation. This adaptive feature is particularly valuable for AA, given that lesion activity—and consequently ROS levels—exhibits dynamic variability between individuals or even within the same individual over time.

#### Immunomodulation beyond local effects

In this study, we found that IMQ can cause splenomegaly in AA mice, which is consistent with the findings of other studies [72]. Concomitantly, H&E staining of spleen sections revealed marked enlargement of the splenic corpuscles in these AA mice, accompanied by extensive inflammatory cell infiltration (Additional file 2), which was likely driven by antigen-specific T-cell activation and inflammatory cell accumulation. Notably, following IMQ withdrawal and hydrogel intervention, the splenic corpuscles gradually regressed in both treatment groups; at the end of the experiment, however, the PVA-TSPBA@miR-665^OE^ hydrogel group presented not only a lower spleen weight but also a smaller number of plenic corpuscles than the hydrogel-only group did, underscoring the superior efficacy of the miR-665-containing treatment.

In addition to promoting local hair regrowth, PVA-TSPBA@miR-665^OE^ hydrogel treatment alleviated splenomegaly more effectively than hydrogel-only treatment did (Additional file 3), a finding that points to potential systemic immunomodulatory effects of locally delivered miR-665. To explain this observed reduction in splenomegaly (an indirect phenotypic readout), we propose two hypothetical interconnected mechanisms (supported by indirect evidence but not directly validated in this study): First, via a hypothesized “local-to-systemic immunomodulatory circuit”: inhibiting aberrant STAT3 activation in lesional tissues may disrupt local IFN-γ-driven inflammatory loops, thereby reducing hair follicle-derived autoantigen presentation and dampening CD8 + T-cell activation [1]—which could in turn diminish T-cell homing to the spleen and alleviate splenomegaly. Second, a speculative remote effect of miRNAs: a small fraction of miR-665 may enter the systemic circulation and be taken up by splenic immune cells to directly inhibit STAT3, further suppressing excessive immune activation. In contrast, the mild improvement in the hydrogel-only group may reflect indirect modulation of the local oxidative stress microenvironment or spontaneous recovery post-IMQ withdrawal [73]; effects that are likely amplified by miR-665’s specific immunomodulatory action.

Taken together, the reduction in splenomegaly and splenic corpuscle size highlights two key implications. First, this finding supports the miR-665-dependent nature of the observed effects, as the most pronounced reductions in splenomegaly were limited to the miR-665 OE group. Second, local treatment can elicit beneficial systemic immunomodulation—a critical advantage for managing AA, which has an underlying systemic immune basis [1].

### Clinical translation and practical utility of the PVA-TSPBA hydrogel in AA

AA, characterized by well-circumscribed hair loss patches, is inherently suited to localized interventions. The injectable PVA-TSPBA hydrogel enables minimally invasive, precise targeting of focal lesions via fine-gauge needles (30G–32G), mirroring the technique for intralesional corticosteroid injections—the current standard for limited AA.

Notably, its shear-thinning and self-healing properties facilitate smooth injection through narrow needles while ensuring retention at the site, enabling spatially controlled miR-665 delivery to the perifollicular inflammatory microenvironment. This maximizes the local drug concentration and minimizes systemic exposure, whereas ROS-responsive release further enhances specificity by preferentially liberating miR-665 in oxidative stress-rich AA lesions. Given the sustained miR-665 release profile (up to 10 days in murine models), 10-day injection intervals are anticipated, reducing treatment frequency versus daily topicals or oral agents to improve compliance. For larger or multiple patches, grid-pattern injection ensures a uniform distribution, and the transparency of the hydrogel is advantageous for clinical monitoring. Future clinical studies should optimize injection techniques, dosages, and intervals in humans, and evaluate combinations with modalities such as microneedling to enhance efficacy in extensive or refractory AA.

This study introduced several key innovations in AA therapy. First, this is the first study to directly compare EMSC-EVs and UMSC-EVs in AA models, demonstrating the superior hair-regenerative efficacy of EMSC-EVs and identifying miR-665 as a critical mediator. Second, we engineered a ROS-responsive PVA-TSPBA hydrogel tailored to the oxidative microenvironment of AA, enabling dual-mode miR-665 release (ROS-triggered burst and hydrolysis-mediated sustained release) via dynamic borate bonds and hydrogen bonding—outperforming nonspecific stimuli-responsive systems. Third, combining miR-665 with this hydrogel resulted in localized, sustained efficacy in our AA mice while reducing potential systemic side effects, which may help to address some limitations of conventional therapies (JAK inhibitors, corticosteroids) and existing RNA delivery systems (e.g., liposomes, viral vectors) in terms of stability and immunogenicity.

## Limitations and future directions

Despite the promising findings of this study, several limitations warrant consideration. First, while the C3H/HeJ mouse model is robust for AA research, it fails to fully recapitulate the heterogeneity of human AA, and translational accuracy could be enhanced by validating findings in mice transplanted with patient-derived AA hair follicles [74]. Second, although systemic immunomodulatory effects of the PVA-TSPBA@miR-665^OE^ hydrogel have been observed, direct evidence for the underlying mechanisms of immunomodulation remains insufficient; key uncertainties include whether low levels of miR-665 enter the systemic circulation, its distribution in splenic immune subsets, and whether its regulation of splenic immunity is mediated by STAT3. Additionally, the hypothesized ROS-dependent “self-regulating” release of miR-665 from the hydrogel in vivo was inferred solely from in vitro H₂O₂ concentration-dependent release data, as we did not directly measure ROS levels in AA lesional skin or quantify miR-665 release kinetics in situ. Third, while the PVA-TSPBA hydrogel relies on ROS (H₂O₂) to trigger miR-665 release, the current study does not directly assess whether exposure to ROS/H₂O₂ during this process induces chemical damage or oxidation of miR-665 itself. Although in vivo RT-qPCR results confirmed retained miR-665 activity in skin lesions on days 3, 6, and 9 post-hydrogel injection, this indirect readout does not exclude the possibility of partial oxidative modification of miR-665—especially under the high H₂O₂ concentrations (e.g., 100 µM) used in in vitro release studies. Notably, we cannot confirm whether miR-665 retains full biological activity under such high oxidative stress, which represents a limitation of the current ROS-responsive design. Finally, the long-term safety profile of hydrogel degradation products is uncharacterized, a 6–12 month chronic toxicity assessment would be valuable to further support potential clinical translation.

To address these gaps, future studies will focus on targeted mechanistic validation, translational assessment, and potential therapeutic optimization, with key efforts including: [1] validating the ROS-dependent “self-regulating” release mechanism by quantifying ROS levels in AA lesional skin and measuring miR-665 release kinetics in situ using imaging-based tracking; [2] verifying systemic immunomodulatory mechanisms by quantifying serum miR-665 in PVA-TSPBA@miR-665^OE^ hydrogel-treated mice at 3, 7, and 10 days postinjection, tracking the spatial distribution of Cy3-labeled miR-665 in splenic immune cells via fluorescence imaging of spleen sections, characterizing changes in splenic T cell subsets and cytokine profiles via flow cytometry, and assessing splenic STAT3 protein levels and phosphorylation status via WB combined with immunohistochemistry; [3] assessing the chemical integrity and biological activity of miR-665 under high H₂O₂ concentrations (e.g., 100 µM) via mass spectrometry and functional assays, ensuring ROS-responsive release does not compromise miR-665’s therapeutic function; and [4] preliminary exploration of synergistic therapeutic strategies—such as codelivery of miR-665 with small molecules or combination with mesodermal therapies proven promising for hair follicle regeneration—building on validated synergistic approaches in other disease contexts [75, 76].

## Conclusion

In summary, this study demonstrated that the delivery of miR-665, via a smart ROS-responsive PVA-TSPBA hydrogel, effectively attenuated inflammatory damage and promoted hair follicle regeneration in AA by targeting the STAT3. The hydrogel system protects miR-665 from rapid degradation and supports ROS-responsive release that is hypothesized to occur preferentially within the inflammatory microenvironment, which may enhance therapeutic precision and durability observed in preclinical models. Our findings highlight the potential of combining miRNA-based gene regulation with advanced biomaterial engineering to develop next-generation therapies for autoimmune and inflammatory conditions. This strategy not only addresses the limitations of current treatments but also opens new avenues for localized and sustained immunomodulation in regenerative dermatology.

## Supplementary Information

Supplementary Material 1

Supplementary Material 2

Supplementary Material 3

## Data Availability

The datasets generated and/or analysed during the current study are not publicly available due to ongoing research projects that rely on these data, and making them public prematurely may disrupt the integrity of future investigations, but are available from the corresponding author on reasonable request.

## Abbreviations

AA: Alopecia areata
IFN-γ: γ-interferon
miRNA: microRNA
EVs: Extracellular vesicles
EMSCs: Embryonic mesenchymal stem cells
UMSCs: Umbilical cord mesenchymal stem cells
EMSC-EVs: Extracellular vesicles derived from human embryonic mesenchymal stem cells
UMSC-EVs: Extracellular vesiclesderived fromhumanumbilical cord mesenchymal stem cell
PVA: Polyvinyl alcohol
TSPBA: N^1^, ^n3^-bis(4-boronobenzyl)-^n1^, ^n1^, ^n3^, ^n3^-tetramethylpropane-1,3-diaminium bromide
JAK-STAT: Janus kinase-signal transducer and activator of transcription
IMQ: Imiquimod
DPCs: Dermal papilla cells
HaCaT cells: Human immortalized keratinocytes
LE: Loading efficiency
BSA: Bovine serum albumin
W/w: Weight/weight
FTIR: Fourier transform infrared spectroscopy
OE: Overexpression
NC: Negative control
KD: Knockdown
RT-qPCR: Reverse transcription-quantitative polymerase chain reaction
WB: Western blotting
ROS: Reactive oxygen species
CCK-8: Cell Counting Kit-8
EdU: 5-ethynyl-2'-deoxyuridine
H&E: Hematoxylin and eosin
SEM: Scanning electron microscope
UTR: Untranslated region
GFP: Green fluorescent protein
PBS: Phosphate-buffered saline
CI: Confidence interval
OD: Optical density
IHC: Immunohistochemistry
