# Supplementary figures and images for: ROS-responsive hydrogel-delivered miR-665 targets STAT3 to alleviate inflammation and promote hair follicle regeneration in alopecia areata
Source: J Nanobiotechnology. 2026 Feb 21;24:285. doi: 10.1186/s12951-026-04214-7 (PMC13032648; doi:10.1186/s12951-026-04214-7)

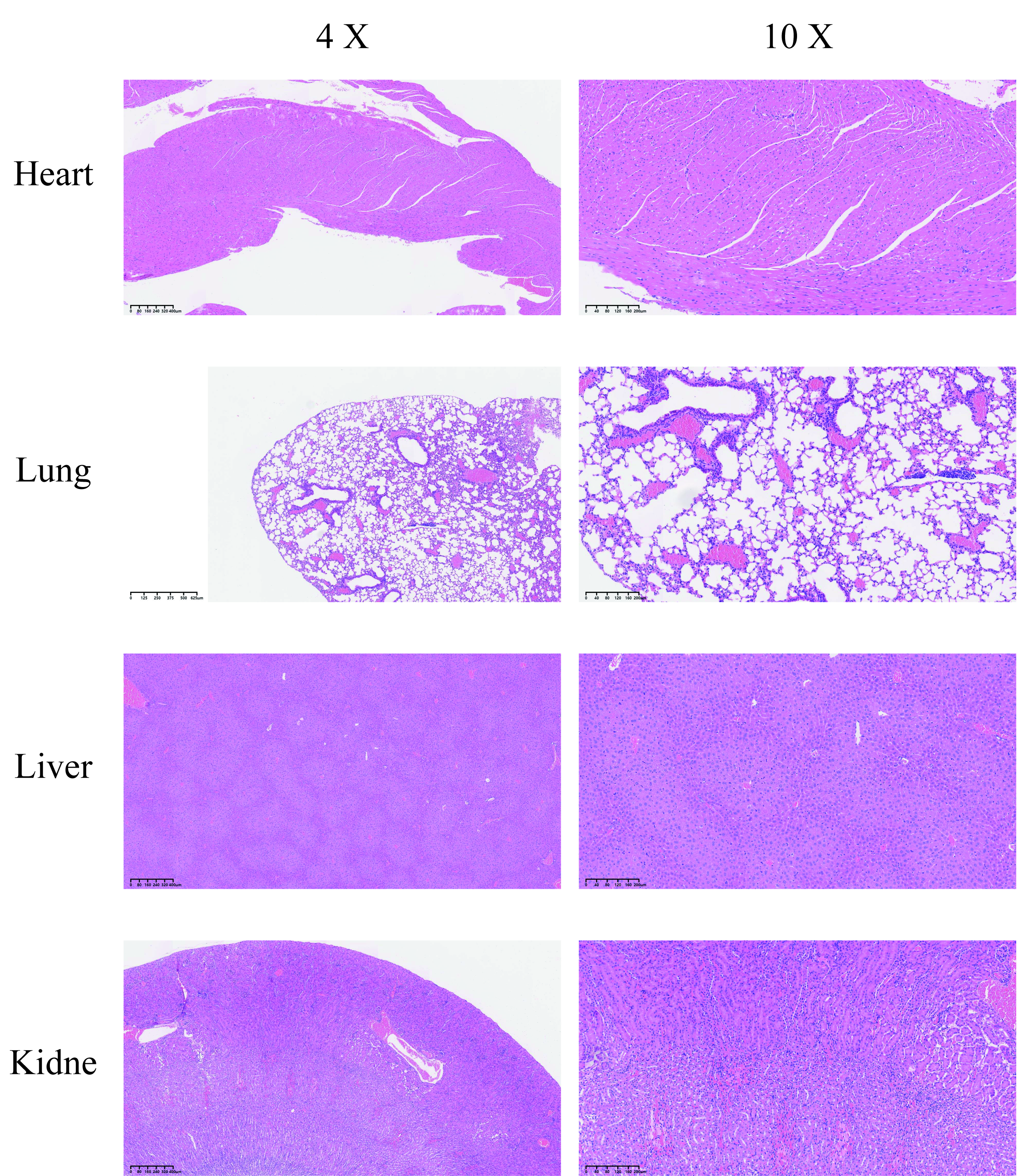

Supplement: Supplementary file 1 — Supplementary Material 1 [file 12951_2026_4214_MOESM1_ESM.tif]

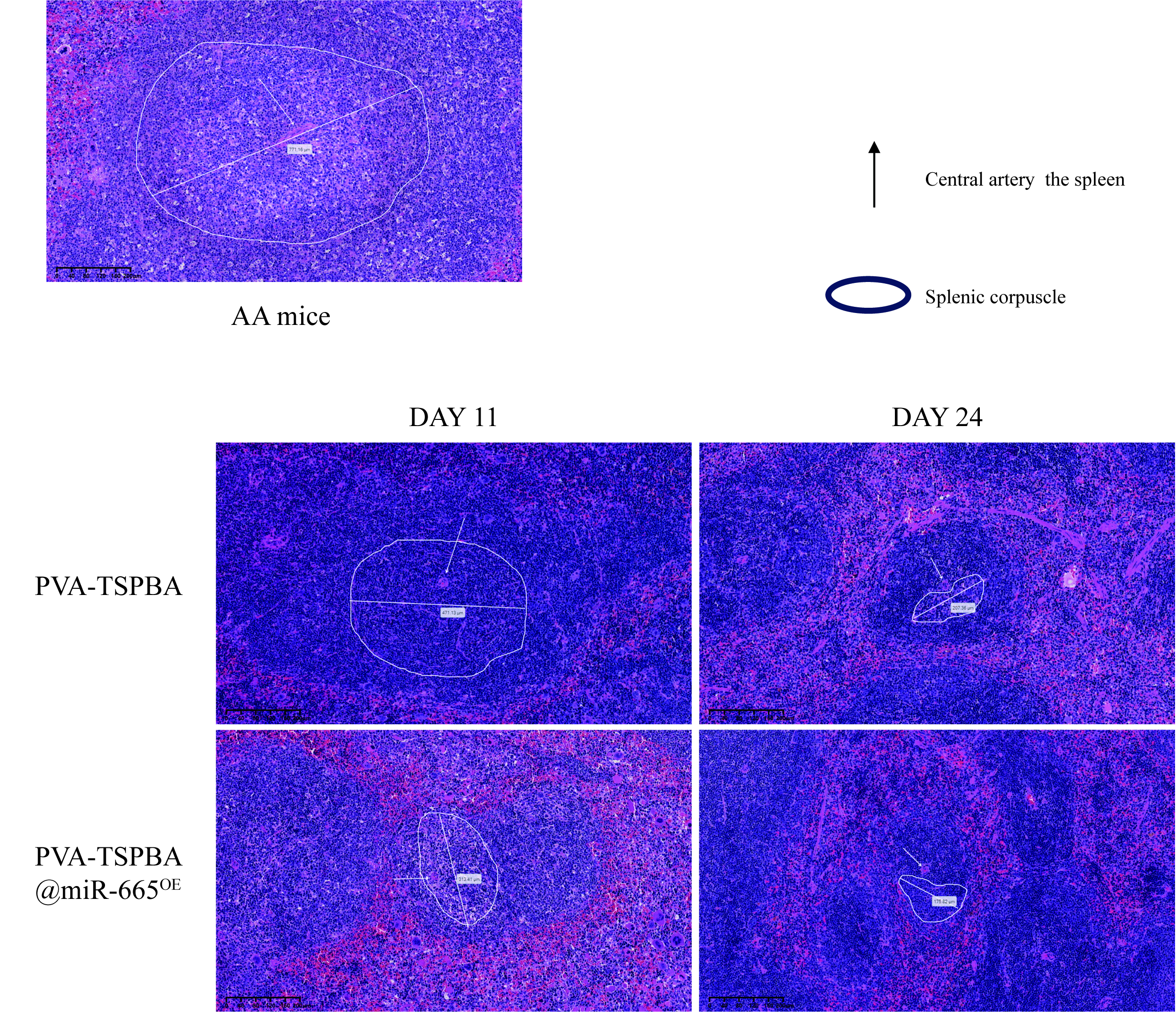

Supplement: Supplementary file 2 — Supplementary Material 2 [file 12951_2026_4214_MOESM2_ESM.tif]

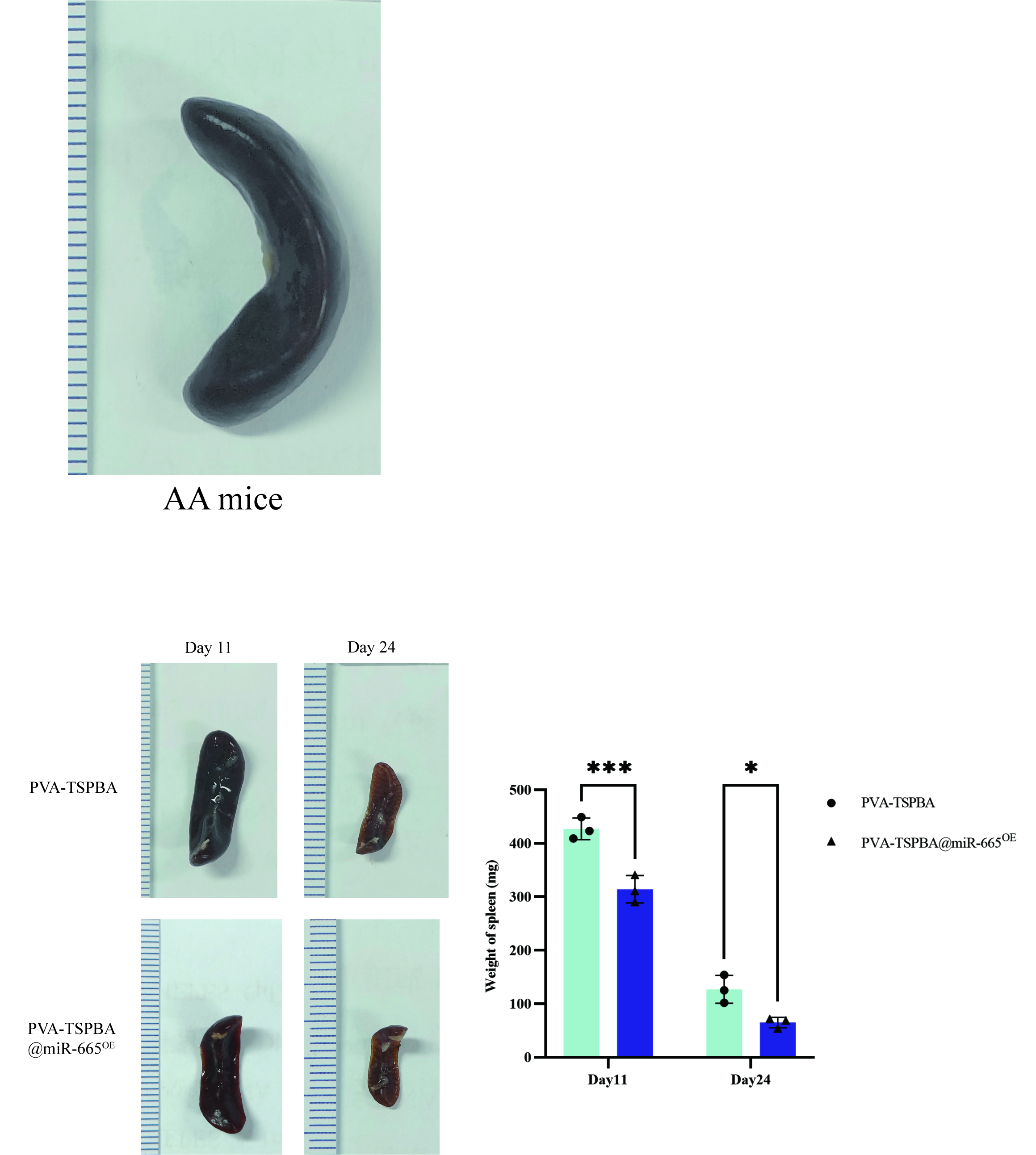

Supplement: Supplementary file 3 — Supplementary Material 3 [file 12951_2026_4214_MOESM3_ESM.tif]
